# CAMSAP2-mediated noncentrosomal microtubule acetylation drives hepatocellular carcinoma metastasis

**DOI:** 10.7150/thno.42596

**Published:** 2020-02-19

**Authors:** Dongxiao Li, Xiangming Ding, Meng Xie, Zheng Huang, Ping Han, Dean Tian, Limin Xia

**Affiliations:** 1 Department of Gastroenterology, Tongji Hospital of Tongji Medical College, Huazhong University of Science and Technology, Wuhan 430030, Hubei Province, China; 2 Institute of Liver and Gastrointestinal Diseases, Tongji Hospital of Tongji Medical College, Huazhong University of Science and Technology, Wuhan 430030, Hubei Province, China

**Keywords:** hepatocellular carcinoma, noncentrosomal microtubule, CAMSAP2, acetylation, HDAC6

## Abstract

**Rationale:** Emerging evidence suggests that noncentrosomal microtubules play an essential role in intracellular transport, cell polarity and cell motility. Whether these noncentrosomal microtubules exist or function in cancer cells remains unclear.

**Methods:** The expression and prognostic values of CAMSAP2 and its functional targets were analyzed by immunohistochemistry in two independent HCC cohorts. Immunofluorescence and co-immunoprecipitation were used for detection of CAMSAP2-decorated noncentrosomal microtubule. Chromatin immunoprecipitation and luciferase report assays were used to determine the c-Jun binding sites in HDAC6 promoter region. *In vitro* migration and invasion assays and *in vivo* orthotopic metastatic models were utilized to investigate invasion and metastasis.

**Results:** We reported a microtubule minus‑end‑targeting protein, CAMSAP2, is significantly upregulated in hepatocellular carcinoma (HCC) and correlated with poor prognosis. CAMSAP2 was specifically deposited on microtubule minus ends to serve as a “seed” for noncentrosomal microtubule outgrowth in HCC cells. Upon depletion of CAMSAP2, the noncentrosomal microtubule array was transformed into a completely radial centrosomal pattern, thereby impairing HCC cell migration and invasion. We further demonstrated that CAMSAP2 cooperates with EB1 to regulate microtubule dynamics and invasive cell migration via Trio/Rac1 signaling. Strikingly, both immunofluorescence staining and western blotting showed that CAMSAP2 depletion strongly reduced the abundance of acetylated microtubules in HCC cells. Our results revealed that HDAC6, a promising target for cancer therapy, was inversely downregulated in HCC and uniquely endowed with tumor-suppressive activity by regulation CAMSAP2-mediated microtubule acetylation. Mechanistically, CAMSAP2 activates c-Jun to induce transrepression of HDAC6 through Trio-dependent Rac1/JNK pathway. Furthermore, NSC23766, a Rac1-specific inhibitor significantly inhibited CAMSAP2-mediated HCC invasion and metastasis.

**Conclusions:** CAMSAP2 is functionally, mechanistically, and clinically oncogenic in HCC. Targeting CAMSAP2-mediated noncentrosomal microtubule acetylation may provide new therapeutic strategies for HCC metastasis.

## Introduction

Hepatocellular carcinoma (HCC) is the sixth most common neoplasm and the third leading cause of cancer-related death worldwide 1. The poor prognosis of patients with HCC mainly results from distant metastasis and tumor recurrence after curative resection, which occur at high frequencies 2. Diverse microtubule-targeting agents have been part of the pharmacopoeia of anticancer therapy for decades; however, they have little effect on HCC metastasis 3.

It is generally accepted that the centrosome is the dominant microtubule-organizing center. Microtubules in interphase cells are typically arrayed in a starburst pattern, with minus ends anchored at the centrosome and plus ends extending radially toward the plasma membrane 4. However, in many cell types—epithelial and neuronal cells—a unique subset of microtubules is untethered from the centrosome and instead distributed throughout the cytoplasm with minus ends free; this subset is required for intracellular transport, cell polarity, and cell motility 5,6. Whether these noncentrosomal microtubules exist or function in cancer cells remains unclear. Therefore, exploring microtubule nucleation and distribution patterns in HCC cells may provide new insights into the suppression of HCC metastasis.

Microtubules are polarized cytoskeletal filaments with two structurally and functionally distinct ends. While the function and dysfunction of microtubule plus-end dynamics and plus-end-tracking proteins (+TIPs)—EB1, CLIP170, and CLASP2—are quite well understood 7, studies on microtubule minus‑end‑targeting proteins—calmodulin‑regulated spectrin‑associated proteins (CAMSAPs) in mammals and Patronin in invertebrates 5,6—have only just begun to emerge. CAMSAP2, a member of the CAMSAP/Nezha/Patronin family, is specifically deposited on microtubule minus ends and serves as a “seed” for noncentrosomal microtubule outgrowth. Depletion of CAMSAP2 results in the transformation of the partially noncentrosomal microtubule network into a completely radial centrosomal array, impeding cell polarization, intracellular transport, and organelle assembly 8,9. Notably, CAMSAP2 depletion impairs directional cell migration 10,11, in line with observations that microtubules are released from the centrosome in migrating cells 12. These findings raise the question of whether migration and invasion in cancer cells are promoted through maintenance of noncentrosomal microtubules and suppression of the microtubule-organizing ability of the centrosome.

Tubulin undergoes several highly conserved posttranslational modifications (PTMs) including detyrosination and further cleavage to Δ2-tubulin, acetylation, polyglutamylation, and polyglycylation 13,14. Tubulin PTMs provide a mechanism for adapting to specific cellular functions by regulating microtubule properties and, therefore, extended the “tubulin code” 15. Acetylated microtubules accumulate at the leading edge, thus promoting directional cell locomotion and chemotaxis 16,17. Therefore, we questioned whether CAMSAP2-mediated noncentrosomal microtubules are subjected to acetylation for promoting HCC cell migration and invasion.

Here, we report a microtubule minus‑end‑targeting protein, CAMSAP2, promotes HCC invasion and metastasis by regulating noncentrosomal microtubule remodeling and acetylation. Investigating the function and mechanism of CAMSAP2-decorated noncentrosomal microtubule in HCC may provide new clues to suppress HCC metastasis.

## Materials and Methods

### Cell lines and culture

Huh7 and MHCC97H cell lines were purchased from the Stem Cell Bank, Chinese Academy of Sciences. HepG2, PLC/PRF/5, SW1990 and SW620 cell lines were obtained from the American Type Culture Collection. All the cell lines were authenticated by short tandem repeat analysis and routinely checked for *Mycoplasma* contamination using the MycoAlert Mycoplasma detection kit. Cells were cultured in Dulbecco's Modified Eagle's Medium (HyClone, UT, USA) containing 10% fetal bovine serum (Gibco, CA, USA), maintained at 37°C in a 5% CO_2_ incubator.

### RNA interference

Cells were transfected with small interfering (si)RNAs (Ribo Bio, Guangzhou, China; siCAMSAP2#1: 5'-GAAACAGTTTAGCCACATA-3' and siCAMSAP2#2: 5'-GAACAACAGTCATGTATCT-3') using Lipofectamine 3000 (Invitrogen, CA, USA) per the manufacturer's instructions. The interference efficacy was verified by western blotting.

### Immunofluorescence (IF) and imaging

Cells were fixed with 4% paraformaldehyde at room temperature for 15 min and, then, permeabilized with phosphate-buffered saline containing 0.2% Triton X-100 for 10 min. For tissue IF, human HCC and corresponding adjacent noncancerous tissues were fixed with 4% paraformaldehyde, paraffin-embedded, and cut into 4-µm-thick sections. After routine dewaxing, rehydration, and antigen retrieval, the cells were permeabilized, blocked with 5% goat serum, and incubated with primary antibodies at 4°C overnight. The cells or tissues were washed with PBS and, then, incubated with the appropriate secondary antibodies. Antibodies are listed in Table S9.

Fluorescence was detected using an Olympus fluorescence microscope equipped with oil-immersion lenses with 100×1.40, 40×0.9, 20×0.75, 10×0.40, or 4×0.16 numerical aperture, and an Olympus laser-scanning confocal microscope equipped with a Plan Apo 60×1.40 numerical aperture oil-immersion lens. Images were processed using Photoshop CS5 (Adobe Systems) and Imaris (Bitplane) software. IF signal intensity was quantified as described previously 11, with slight modifications. IF signal intensity distribution was measured using the ImageJ Radial Profile plugin: a circle with the indicated radius was drawn at the center of gamma-tubulin, the Golgi complex, or the nucleus, and the signal intensity along the radius was measured Fluorescence intensities were normalized to the maximum intensity of each cell.

Other protocols used in this study are described in the Supplementary Materials.

## Results

### CAMSAP2 is significantly upregulated in HCC tissues and indicates a poor prognosis

We first investigated the expression of CAMSAP family members in different publicly available liver cancer datasets. The Cancer Genome Atlas dataset (TCGA) revealed that mRNA levels of the CAMSAP family were significantly increased in liver cancer specimens when compared to the levels in normal liver tissues (Figure S1A). Immunohistochemistry (IHC) tissue microarray data from Human Protein Atlas program database revealed high or medium CAMSAP2 staining intensity in 10 out of 12 liver cancer samples, whereas only 3 out of 12 cases showed medium staining of CAMSAP1 and CAMSAP3 (Figure S1B). Kaplan-Meier analysis based on TCGA data revealed that liver cancer patients with high CAMSAP2 mRNA levels had a significantly shorter overall survival (OS) and disease-free survival (DFS) than those who with low CAMSAP2 mRNA levels (Figure S1C). There was no obvious correlation between poor patient outcome and high expression of CAMSAP1 or CAMSAP3 (Figure S1C). Moreover, the increased mRNA expression of CAMSAP2 also observed in pancreatic adenocarcinoma (PAAD), stomach adenocarcinoma (STAD) and colon adenocarcinoma (COAD) tissues based on TCGA data (Figure S1D). Kaplan-Meier analysis based on TCGA dataset revealed that PAAD, STAD and COAD patients with high levels of CAMSAP2 mRNA had a shorter OS and DFS than those with low mRNA expression of CAMSAP2 (Figure S1E). Collectively, these findings suggested that CAMSAP2 may serve as a candidate biomarker for HCC prognosis.

**Figure 1:**
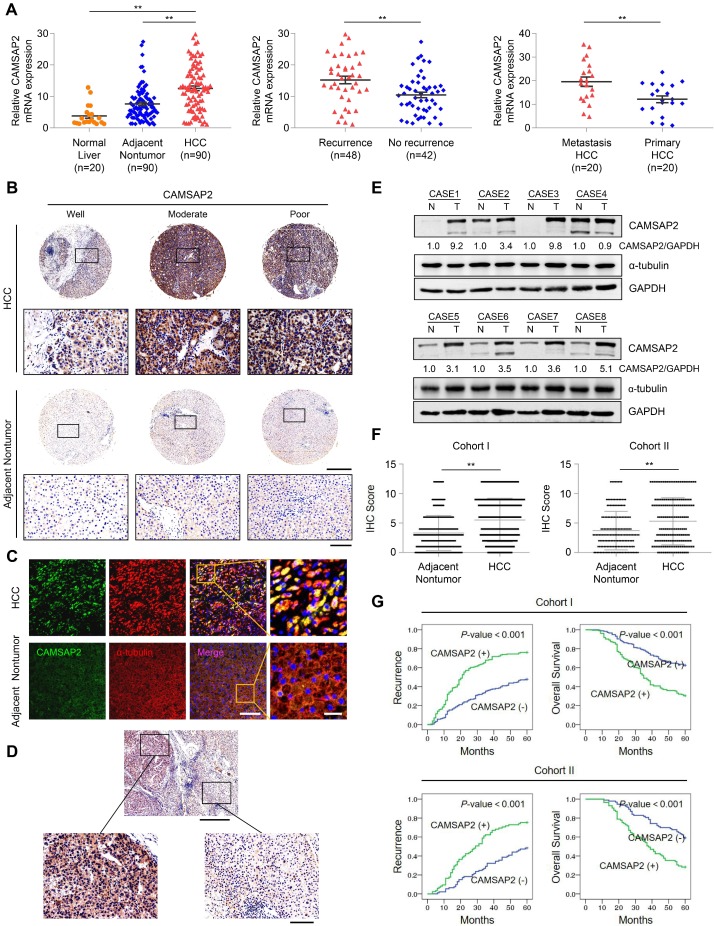
** CAMSAP2 is significantly upregulated in HCC tissues and indicates a poor prognosis.** (A) Relative *CAMSAP2* mRNA levels in 20 normal liver samples and 90 paired HCC and adjacent nontumorous samples (Left). Relative *CAMSAP2* mRNA levels in HCC patients with (n = 48) or without (n = 42) recurrence (Middle). Relative *CAMSAP2* mRNA levels in 20 paired primary and metastatic HCC tissues (Right). **P < 0.01. (B) Representative IHC staining images showing CAMSAP2 expression in HCC and adjacent nontumorous tissues. Scale bars, 500 µm (upper), 100 µm (lower). (C) Representative IF staining images of CAMSAP2 (green), α-tubulin (red), and DNA (DAPI, blue) in HCC and matched adjacent nontumorous tissues. Scale bars, 50 µm (left), 100 µm (right). (D) Representative IHC staining images of CAMSAP2 in HCC and peritumoral area. Scale bars, 500 µm (upper), 100 µm (lower). (E) CAMSAP2 protein levels in 8 paired HCC and adjacent nontumorous tissues as detected by western blotting. N, adjacent nontumorous tissue; T, tumor tissue. (F) IHC scores of CAMSAP2 in two independent cohorts of human HCC (cohort I, n = 360; cohort II, n = 178). (G) Kaplan-Meier analysis was used to determine the correlation between CAMSAP2 expression and recurrence or OS in two independent cohorts.

We quantified CAMSAP2 expression in 90 pairs of HCC and adjacent nontumorous tissue samples and 20 normal liver tissues using quantitative reverse-transcription polymerase chain reaction (RT-q)PCR. HCC tissues displayed marked upregulation of CAMSAP2 mRNA, compared with adjacent nontumorous and normal liver tissues (Figure 1A). CAMSAP2 mRNA expression was higher in HCC tissues from patients with recurrence than in those from patients without recurrence (Figure 1A). CAMSAP2 mRNA expression was significantly upregulated in metastatic HCC tissues, compared to matched primary HCC tissues (Figure 1A). IHC, immunofluorescence (IF), and western blot analyses revealed that CAMSAP2 protein expression was upregulated in HCC compared with adjacent noncancerous tissues (Figure 1B-E). Intriguingly, IF staining revealed symmetrical staining of CAMSAP2 and α-tubulin throughout the cytoplasm in nontumor tissues, whereas highly asymmetrical signals were detected in HCC tissues (Figure 1C).

To investigate the potential role of CAMSAP2 in determining clinical outcomes for HCC, we analyzed its expression in two independent cohorts of human HCC (cohort I, n = 360; cohort II, n = 178) by IHC. CAMSAP2 was dramatically upregulated in HCC compared with adjacent nontumor tissues (Figure 1F) (Table S1). Positive and negative controls are shown in Figure S3B. In addition, IHC staining confirmed that CAMSAP2 was also upregulated in PAAD, STAD and COAD cancer specimens compared with adjacent nontumor tissues (Figure S1F). Analysis of the clinicopathological characteristics revealed that CAMSAP2 overexpression was strongly associated with multiple tumors, increased tumor size, microvascular invasion, poor tumor differentiation, and a higher tumor-nodule-metastasis stage (Table S1). HCC patients with high CAMSAP2 expression had a higher chance of recurrence and a shorter OS than those with low CAMSAP2 expression (Figure 1G). Multivariate analysis identified CAMSAP2 expression as an independent predictor of postoperative recurrence and OS (Table S2 and S3). Together, these data indicated that CAMSAP2 is a potential prognostic biomarker for malignant progression and metastasis of HCC.

### CAMSAP2 promotes HCC invasion and metastasis *in vitro* and *in vivo*

**Figure 2:**
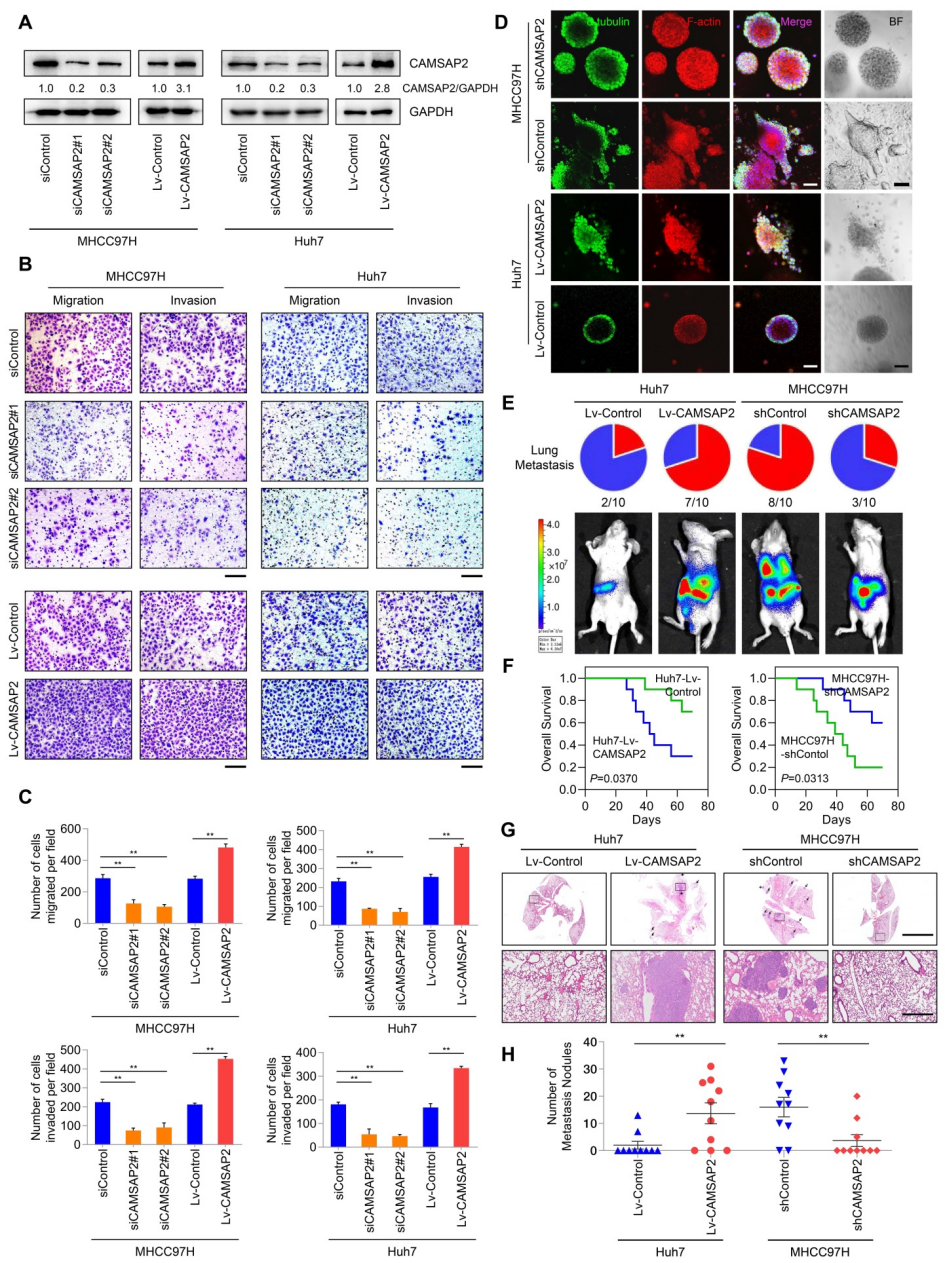
** CAMSAP2 promotes HCC invasion and metastasis *in vitro* and *in vivo.***(A) Western blot analysis of CAMSAP2 in the indicated transfected HCC cells. (B) Transwell assays of the indicated HCC cells. Migrating and invading cells were quantified in (C). Data are the mean ± SEM from triplicate experiments. **P < 0.01. Scale bar, 400 μm. (D) IF staining of ɑ-tubulin (green), F-actin (red), and DNA (DAPI, blue) to monitor three-dimensional invasion morphology. Scale bar, 100 μm. (E) Incidence of lung metastasis and bioluminescence imaging of each group at 10 weeks after orthotopic xenografting with the indicated HCC cells. (F) OS of mice in the different groups. (G) Representative H&E staining of lung tissues from each group. Scale bars, 500 mm (upper), 500 µm (lower). (H) Number of metastatic lung nodules observed in each group. **P < 0.01.

For loss- and gain-of-function analyses, we knocked down and overexpressed CAMSAP2 in MHCC97H and Huh7 cells, denoted as “MHCC97H/Huh7-siCAMSAP2” and “MHCC97H/Huh7-Lv-CAMSAP2,” and we evaluated CAMSAP2 expression by western blotting (Figure 2A). CAMSAP2 depletion markedly reduced HCC cell migration and invasion in MHCC97H and Huh7 cells, whereas CAMSAP2 upregulation had the opposite effect (Figure 2B-C). Further, the decreased migration and invasion capabilities also confirmed in CAMSAP2-knockout MHCC97H cell line (Figure S2A-C).

To mimic the *in-vivo* invasion phenotype, we used three-dimensional cultures to visualize tumor sphere invasion. Because of its transient effects, small interfering (si)RNA-mediated knockdown cannot be used in three-dimensional culture. Instead, we used a lentiviral vector to establish two CAMSAP2-knockdown cell lines, denoted as “MHCC97H-shCAMSAP2” and “Huh7-shCAMSAP2.” IF staining indicated that MHCC97H cells with high metastatic potential showed an obvious invasion phenotype, whereas MHCC97H-shCAMSAP2 cells formed a spheroidal tumor nest with well-defined borders (Figure 2D). Opposite results were found in CAMSAP2-overexpressing Huh7 compared with control cells (Figure 2D). These results suggested that CAMSAP2 promotes HCC cell invasion in an *in-vivo*-like environment.

To assess the effect of CAMSAP2 on HCC metastasis *in vivo*, CAMSAP2-overexpressing, -knockdown, and control HCC cells were injected into the livers of nude mice to establish an orthotopic metastatic model. Bioluminescence imaging of the HCC cells prior to orthotopic implantation is shown in Figure S3A. Consistent with the *in-vitro* results, CAMSAP2 upregulation significantly increased intrahepatic and lung metastases, resulting in a shorter OS time of the Huh7-Lv-CAMSAP2 compared with the control group (Figure 2E-F). Hematoxylin and eosin (H&E) staining confirmed that CAMSAP2 overexpression significantly increased the incidence of lung metastasis and the number of metastatic pulmonary nodules (Figure 2G-H). In contrast, CAMSAP2 knockdown reduced the incidence of lung metastasis and the number of metastatic pulmonary nodules, resulting in a longer OS time (Figure 2G-H). Collectively, these findings suggested that CAMSAP2 significantly promotes HCC metastasis.

### CAMSAP2-mediated noncentrosomal microtubule remodeling contributes to directional cell migration in HCC cells

**Figure 3:**
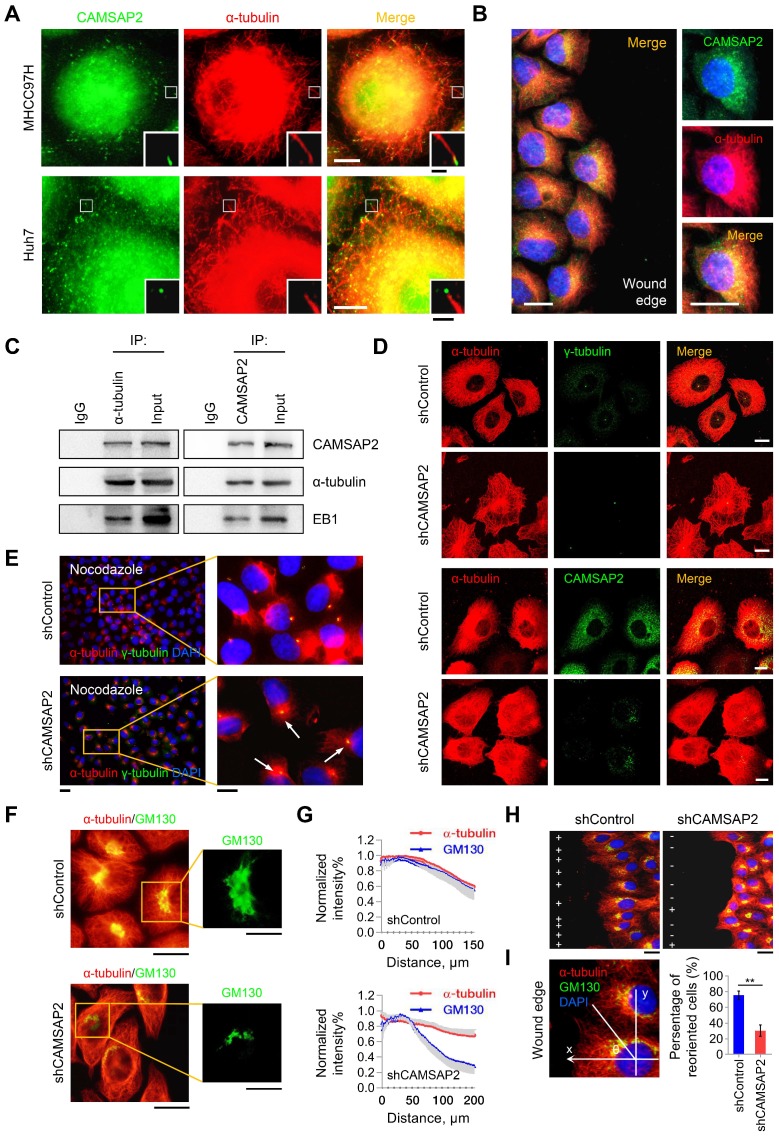
** CAMSAP2-mediated noncentrosomal microtubule remodeling contributes to directional cell migration in HCC cells.** (A) IF staining of CAMSAP2 (green) and α-tubulin (red) in MHCC97H and Huh7 cells. Scale bars, 100 µm, 20 µm (insert). (B) IF staining of CAMSAP2 (green), α-tubulin (red), and DNA (DAPI, blue) in the wound-healing edge of MHCC97H cells. Scale bars, 50 µm. (C) Endogenous co-immunoprecipitation assays revealed that CAMSAP2 interacts with α-tubulin and EB1 in MHCC97H cells. IgG was used as a negative control. (D) Double IF staining of α-tubulin (red) and γ-tubulin/CAMSAP2 (green) in control and MHCC97H-shCAMSAP2 cells. Scale bar, 100 µm. (E) Cells were treated with 15 µM nocodazole at 4°C for 30 min, incubated min at 37°C for another 10 min after drug washout, fixed, and immunostained for α-tubulin (red) and γ-tubulin (green). Scale bars, 500 µm (left), 200 µm (right). (F) Double immunostaining for GM130 (green) and α-tubulin (red) in control and CAMSAP2-knockdown cells. Scale bars, 200 µm (left), 100 µm (right). (G) Quantification of CAMSAP2 and GM130 distribution in CAMSAP2-depleted and control cells. Distance from the center of GM130 is shown on the horizontal axis. Twenty cells were analyzed per group. Intensities were normalized to the maximum intensity of each cell. Data are the mean ± SEM from triplicate experiments. (H) Reorientation of the Golgi apparatus towards the direction of cell migration after 12 h of monolayer wound healing in control and MHCC97H-shCAMSAP2 cells. IF staining of α-tubulin (red), the GM130 (green), and DNA (DAPI, blue). Scale bar, 100 µm. (I) Quantification of directional cell migration in control and MHCC97H-shCAMSAP2 cells. A cell was considered reoriented (+) when GM130 was positioned in the 120° arc towards the wound edge. Approximately 120-150 cells were analyzed for each condition. Data are the mean ± SEM from triplicate experiments. *P < 0.05.

CAMSAP2-decorated noncentrosomal microtubules have been observed in various cell types, including fibroblasts, epithelial cells, and neurons 5,6. Therefore, we questioned whether CAMSAP-decorated noncentrosomal microtubule stretches exist and function in HCC cells. Double IF staining of CAMSAP2 and α-tubulin in MHCC97H and Huh7 cells yielded stretched or punctate CAMSAP2 signals scattered throughout the cytoplasm (Figure 3A). Closer observation revealed that each CAMSAP2 cluster capped a microtubule minus end (Figure 3A). Similar observations were made in HepG2 and PLC/ PRF/5 HCC cell lines (Figure S4A). During wound-induced migration, CAMSAP2 reorganized towards the direction of migration along the tubulin-based protrusion (Figure 3B). Endogenous co-immunoprecipitation assays revealed that CAMSAP2 could be immunoprecipitated with α-tubulin antibody in MHCC97H cells. Similarly, α-tubulin was detected in CAMSAP2 immunoprecipitates (Figure 3C). Additionally, EB1, a +TIP, was also detected in the immunoprecipitates (Figure 3C). These results indicated that CAMSAP2 caps microtubule minus ends and forms CAMSAP-decorated noncentrosomal microtubule stretches in HCC cells.

CAMSAP2 depletion alters the microtubule assembly pattern and, thus, the distribution of microtubule networks. Therefore, we explored the effect of CAMSAP2 depletion on microtubule assembly in HCC cells. Immunostaining revealed that CAMSAP2 depletion transformed the noncentrosomal microtubule network into a symmetrical radial pattern wherein the majority of microtubules nucleated from the centrosome in MHCC97H cells (Figure 3D). Similar observations were made in Huh7 cells (Figure S4B). Next, we treated subconfluent HCC cells with nocodazole for 30 min to completely depolymerize microtubules and, then, observed microtubule repolymerization after nocodazole washout. IF staining revealed that the initial repolymerization microtubules radiated from the centrosome 15 min after drug washout in CAMSAP2-depleted cells, whereas noncentrosomal microtubules were not observed (Figure 3E). These observations implied that CAMSAP2 works to maintain noncentrosomal microtubules, while suppressing the microtubule-nucleating ability of the centrosome in HCC cells.

Next, we investigated the consequences of the altered microtubule pattern on directional cell migration. Maintenance of the Golgi ribbon is crucial for polarized cell migration 10,11. Intriguingly, while protein expression of the Golgi marker GM130 was not altered (Figure S4C), the Golgi apparatus lost its typical ribbon-like morphology in CAMSAP2-depleted MHCC97H cells. The microtubule system became more radial and failed to colocalize with the Golgi stacks (Figure 3F-G). During wound-induced migration, CAMSAP2-depleted HCC cells failed to polarize properly because of defective Golgi reorientation towards the wound edge (Figure 3H-I). Collectively, these results suggested that CAMSAP2 is essential for noncentrosomal microtubule organization, which is required for HCC cell polarization and migration.

### CAMSAP2 cooperates with EB1 to regulate microtubule dynamics and invasive cell migration via Trio/Rac1 signaling

**Figure 4:**
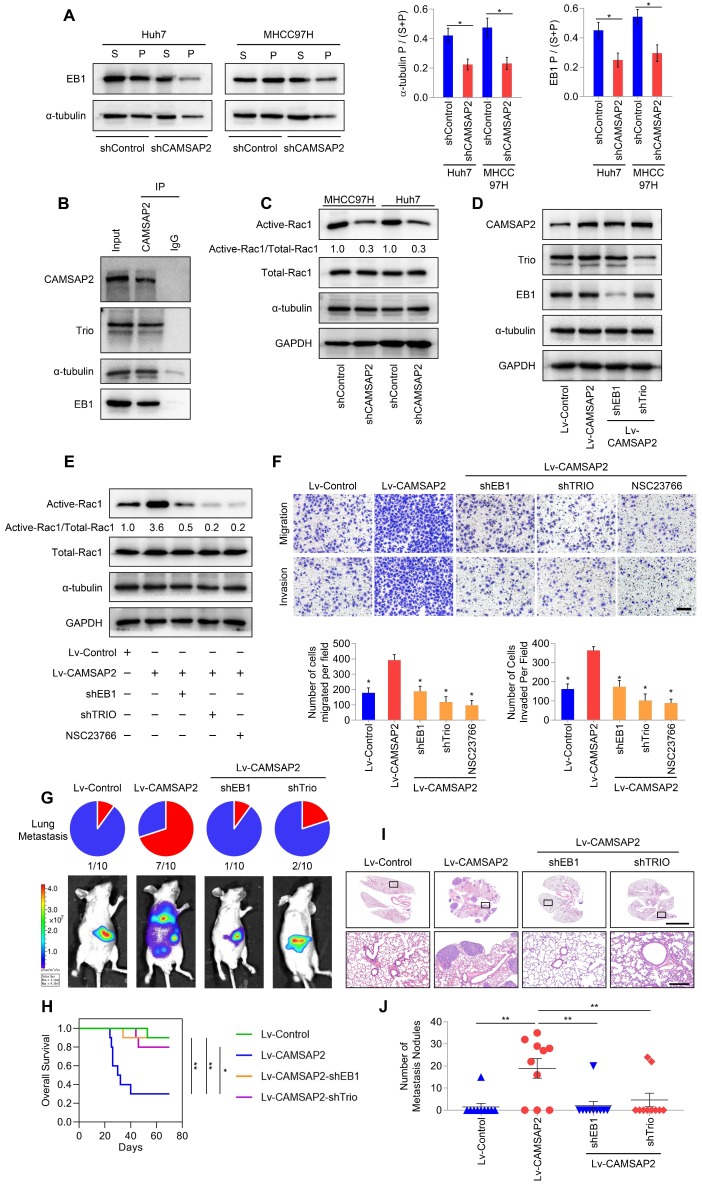
** CAMSAP2 cooperates with EB1 to regulate microtubule dynamics and invasive cell migration via Trio/Rac1 signaling.** (A) Microtubule fractionation was performed to detect depolymerized (soluble, S) and polymerized (pelleted, P) fractions of α-tubulin and EB1. The ratio of polymerized ɑ-tubulin/EB1 to the total amount is presented in the right panel. Data are the mean ± SEM from triplicate experiments. *P < 0.05. (B) Endogenous co-immunoprecipitation assays revealed that CAMSAP2 interacts with Trio, α-tubulin, and EB1 in HCC cells. IgG was used as a negative control. (C) Western blot analysis of active-Rac1, total Rac1, and α-tubulin in the indicated HCC cells. (D) Western blot analysis of CAMSAP2, EB1, and Trio in the indicated transfected HCC cells. (E) Western blot analysis of active-Rac1, total Rac1, and α-tubulin in the indicated HCC cells. NSC23766, a specific inhibitor was used to inactivate Rac1. (F) Transwell assay of the indicated HCC cells. Migrating and invading cells were quantified in the lower panel. Data are the mean ± SEM from triplicate experiments. Scale bar, 400 μm. *P < 0.05. (G) Incidence of lung metastasis and bioluminescence imaging of each group at 10 weeks after orthotopic xenografting with the indicated HCC cells. (H) OS of mice in the different groups. (I) Representative H&E staining of lung tissues from each group. Scale bars, 500 mm (upper), 500 µm (lower). (J) Number of metastatic lung nodules observed in each group. *P < 0.05, **P < 0.01.

We further investigated the regulatory roles of CAMSAP2 in microtubule dynamics by monitoring the behavior of EB1, which autonomously tracks the growing microtubule end and recruits multiple +TIPs to couple the microtubule dynamics to specific cellular events 7,18. IF staining for EB1 in HCC cells produced stretched or punctate signals scattered through the cytoplasm. Closer observation revealed that EB1 cluster capped a microtubule end (Figure S4F). Microtubule fractionation assays revealed that microtubule-associated EB1 was reduced after CAMSAP2 knockdown (Figure 4A). The polymerized-to-total a-tubulin ratio was also decreased in CAMSAP2-knockdown HCC cells (Figure 4A). These findings implied that loss of CAMSAP2 led to a reduction in the amount of polymerized a-tubulin as well as growing microtubule ends.

Microtubule plus ends function as concentration devices for signaling molecules—Rho GTPase guanine nucleotide exchange factors (GEFs) and kinases 7. EB1 at growing microtubule ends promotes the formation of a complex with the GEF triple functional domain protein (Trio), leading to Trio-dependent activation of Rac1 19. Co-immunoprecipitation assays indicated that CAMSAP2 and a-tubulin directly interacted with EB1 and Trio in HCC cells (Figure 4B). Then, we examined Rac1 activity after CAMSAP2 depletion. Western blot analysis revealed that active Rac1 was markedly reduced in CAMSAP2-knockdown HCC cells (Figure 4C). EB1 and Trio knockdown or treatment with the specific Rac1 inhibitor NSC23766 significantly ameliorated the increased expression of GTP-Rac1 (Figure 4D-E) and markedly suppressed the increased migration and invasion capabilities (Figure 4F) induced by CAMSAP2 upregulation.

To determine the role of the EB1/Trio complex in CAMSAP2-mediated HCC metastasis *in vivo*, we established an orthotopic metastatic model. Bioluminescence imaging of the indicated HCC cells prior to orthotopic implantation is shown in Figure S3A. Consistent with the *in-vitro* results, EB1 or Trio knockdown markedly lowered the incidence of lung metastasis and prolonged the OS time in the Huh7-Lv-CAMSAP2 group (Figure 4G-H). Histological analysis confirmed that depletion of EB1 or Trio abrogated lung metastasis induced by CAMSAP2 overexpression (Figure 4I-J). Together, these results suggested that CAMSAP2 cooperates with the EB1/Trio complex to direct microtubule dynamics to invasive cell migration by promoting Rac1 signaling.

### Prognostic significance of the correlation between CAMSAP2 and EB1 or Trio expression in HCC tissues

**Figure 5:**
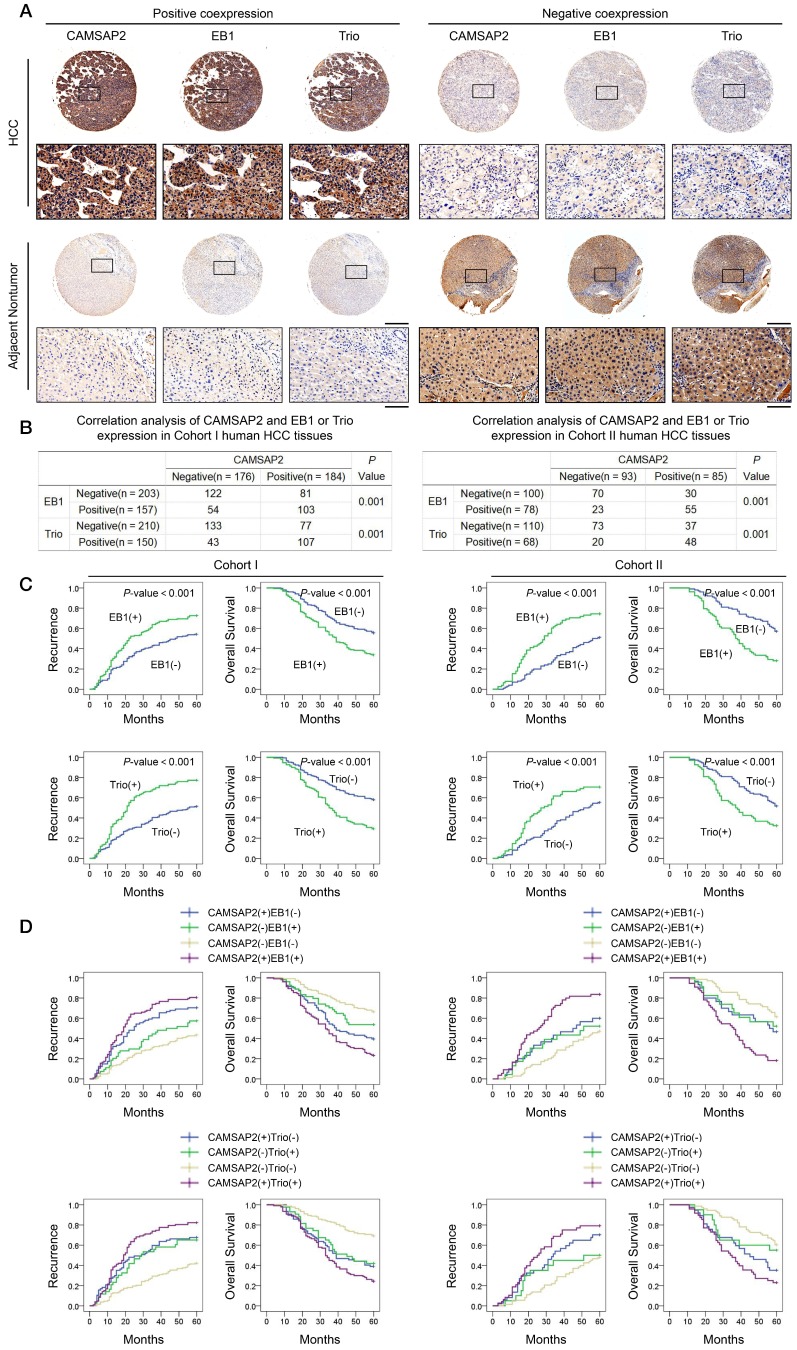
** Prognostic significance of the correlation between CAMSAP2 and EB1 or Trio expression in HCC tissues.** (A) Representative IHC images of CAMSAP2, EB1, and Trio expression in HCC and adjacent nontumorous tissues. Scale bars, 500 µm (upper), 100 µm (lower). (B) Relationship between CAMSAP2 expression and EB1 or Trio expression in two independent cohorts. (C) Kaplan-Meier analysis was used to determine the correlation between EB1 or Trio expression and recurrence or OS in the two cohorts. (D) Kaplan-Meier analysis of the correlation between CAMSAP2/EB1 or CAMSAP2/Trio coexpression and recurrence and OS in the two cohorts.

Clinical associations between CAMSAP2 and EB1 or Trio expression were evaluated in two independent cohorts of HCC patients. IHC revealed that CAMSAP2 overexpression was positively correlated with EB1 and Trio expression (Figure 5A-B). Positive and negative controls are shown in Figure S3B. Analysis of the clinicopathological characteristics in paired HCC tissues showed that upregulation of either EB1 or Trio was significantly correlated with malignant tumor progression and poor prognosis (Figure 5C) (Tables S4 and S5). Further, coexpression of either CAMSAP2/EB1 or CAMSAP2/Trio was associated with the highest recurrence rate and lowest OS in both HCC cohorts (Figure 5D).

### CAMSAP2 promotes microtubule acetylation to control invasive cell migration via an inhibitory interaction with HDAC6

**Figure 6:**
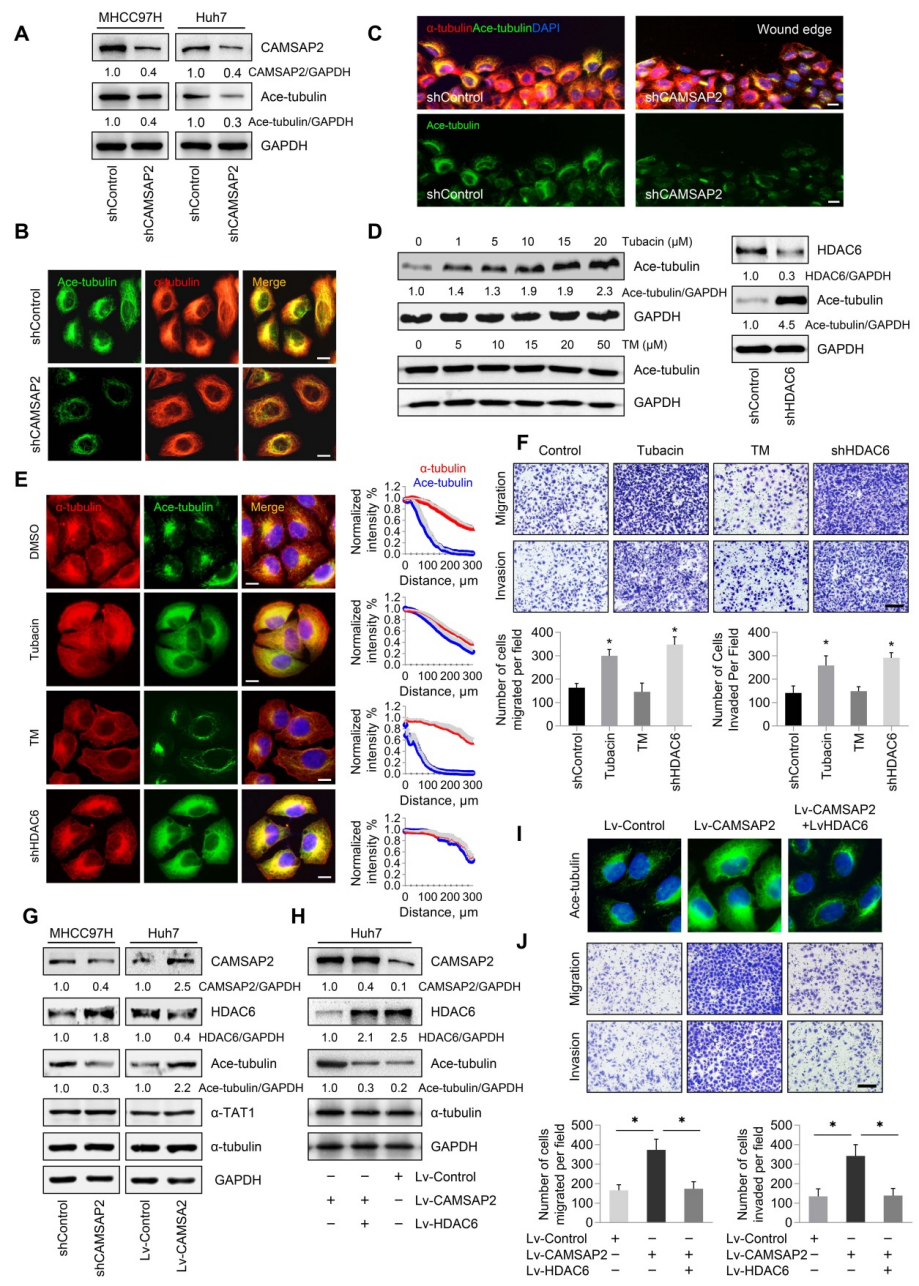
** CAMSAP2 promotes microtubule acetylation to control invasive cell migration via an inhibitory interaction with HDAC6.** (A) Western blot analysis of CAMSAP2 and acetylated α-tubulin in the indicated cells. Ace-tubulin, acetylated α-tubulin. (B) IF staining of α-tubulin (red) and Ace-tubulin (green) in control and MHCC97H-shCAMSAP2 cells. Scale bars, 200 µm. (C) IF staining of α-tubulin (red), Ace-tubulin (green), and DNA (DAPI, blue) in indicated cells at the edge of directional migration. Scale bars, 100 µm. (D) Western blot analysis of Ace-tubulin in the indicated HCC cells. Tubacin and thiomyristoyl (TM) were used to inactivate HDAC6 and sirtuin 2, respectively. (E) IF staining of α-tubulin (red), Ace-tubulin (green), and DNA (DAPI, blue) in the indicated HCC cells. Scale bars, 200 µm. Quantification of α-tubulin and Ace-tubulin distribution in the indicated HCC cells. Distance from the microtubule-organizing center is displayed on the horizontal axis. Intensities were normalized to the maximum intensity of each cell. Twenty-five cells were analyzed for each condition. Data are the mean ± SEM from triplicate experiments. (F) Transwell assays of the indicated HCC cells. Migrating and invading cells were quantified in the lower panel. Data are the mean ± SEM from triplicate experiments. *P < 0.05. Scale bar, 400 μm. (G) Protein levels of CAMSAP2, HDAC6, Ace-tubulin and αTAT1 in the indicated cells as determined by western blotting. (H) Western blot analysis of CAMSAP2, HDAC6, and Ace-tubulin in the indicated HCC cells. (I) IF staining of Ace-tubulin (green) in the indicated cells. Scale bars, 50 µm. (J) Transwell assays of the indicated HCC cells. Migrating and invading cells were quantified in the right panel. Data are the mean ± SEM from triplicate experiments. **P < 0.01. Scale bar, 400 μm (left).

Tubulin PTMs provide a mechanism for microtubules to adapt to specific cellular functions 20. Acetylated microtubules accumulate at the leading edge, thus promoting directional cell locomotion and chemotaxis 16,17. Therefore, we questioned whether CAMSAP2-mediated noncentrosomal microtubules are subjected to acetylation for promoting HCC cell migration and invasion. Western blot analysis revealed that microtubule acetylation was markedly reduced in CAMSAP2-knockdown HCC cells (Figure 6A, Figure S4D). However, there were no obvious changes in the expression of detyrosinated or tyrosinated α-tubulin after downregulation of CAMSAP2 (Figure S5A). Conversely, CAMSAP2 overexpression dramatically elevated α-tubulin acetylation in MHCC97H and Huh7 cells (Figure S4E). The decreased expression of tubulin acetylation and the suppressed migration and invasion capabilities also confirmed in CAMSAP2 knockout HCC cell line as well as SW1990 and SW620 CAMSAP2 knockdown cells lines (Figure S2A-C, Figure S6A-B). Intriguingly, in CAMSAP2-knockdown cells, the polarized acetylated microtubule network was no longer asymmetrical, but showed a symmetrical radial pattern, as revealed by co-immunostaining (Figure 6B). IF staining revealed that the acetylated microtubule network, which typically extends towards the direction of cell migration, was disrupted in migrating CAMSAP2-knockdown HCC cells (Figure 6C). Acetylation of a canonical α-tubulin site has been identified on lysine 40, and α-tubulin point mutations at lysine 40 to arginine (K40R) abrogate tubulin acetylation. To further confirm the role of tubulin acetylation in CAMSAP2-mediated migration and invasion of HCC cells, Huh7-Lv-CAMSAP2 cells were transfected with tubulin-K40R mutant or WT plasmid. Western blot analysis revealed that tubulin-K40R markedly ameliorated the increased expression of microtubule acetylation induced by CAMSAP2 overexpression (Figure S6C). The elevated migration and invasion of CAMSAP2-overexpressing HCC cells were significantly abrogated after K40R mutation. (Figure S6D).

Microtubule acetylation is tightly controlled by the enzymatic activities of multiple acetyltransferases and is negatively regulated through the activities of the deacetylases HDAC6 and to a lesser extent, sirtuin 2 16,17. Therefore, we hypothesized that CAMSAP2 may promote microtubule acetylation by either activating acetyltransferases or inhibiting deacetylases. Inhibition of HDAC6 activity by the specific inhibitor tubacin or HDAC6 depletion enhanced microtubule acetylation in HCC cells (Figure 6D). Acetylated microtubule extended towards the leading edge after HDAC6 inactivation or depletion compared with that in control cells (Figure 6E). However, suppression of sirtuin 2, the only other microtubule deacetylase identified, by treatment with the specific inhibitor thiomyristoyl had no significant effect on α-tubulin acetylation (Figure 6D-E). Inactivation or downregulation of HDAC6 dramatically increased the migration and invasion capabilities of HCC cells (Figure 6F). Thus, HDAC6 was identified as the predominant α-tubulin deacetylase in HCC cells. In addition, suppression of tubulin detyrosination by treatment with the inhibitor PTL had no significant effect on HCC cell migration and invasion (Figure S5B-C).

To validate the deacetylation role of HDAC6 in CAMSAP2-mediated noncentrosomal microtubule acetylation, we examined HDAC6 expression in transfected HCC cells. Both western blotting and real-time PCR analysis showed that CAMSAP2 knockdown in MHCC97H cells significantly increased HDAC6 expression, and that HDAC6 was markedly downregulated in CAMSAP2-overexpressing Huh7 cells with no apparent change in the expression of αTAT1 in the transfected HCC cells (Figure 6G, Figure S7A). The increased expression of HDAC6 also confirmed in CAMSAP2 knockout HCC cell line (Figure S2B). Overexpression of HDAC6 abrogated the increase in microtubule acetylation induced by CAMSAP2 overexpression, suggesting that CAMSAP2-mediated microtubule acetylation is likely due to dysfunctional HDAC6 rather than the activation of acetyltransferases (Figure 6H-I). Upregulation of HDAC6 significantly ameliorated the enhanced migration and invasion capabilities of CAMSAP2-overexpressing Huh7 cells (Figure 6J).

To determine the effect of HDAC6 on CAMSAP2-mediated metastasis *in vivo,* we established an orthotopic metastatic model. HDAC6 down-regulation markedly increased the incidence of lung metastasis and decreased the OS time of the MHCC97H-shCAMSAP2 group compared with that in the control group (Figure S7B-C). Histological analysis further confirmed that the incidence of lung metastasis and the number of metastatic lung nodules in the MHCC97H-shCAMSAP2-shHDAC6 group were significantly increased (Figure S7D-E). Collectively, these results suggested that CAMSAP2 promotes microtubule acetylation to control invasive cell migration via an inhibitory interaction with HDAC6.

### Prognostic significance of the correlation between CAMSAP2 and acetylated α-tubulin or HDAC6 expression in HCC tissues

**Figure 7:**
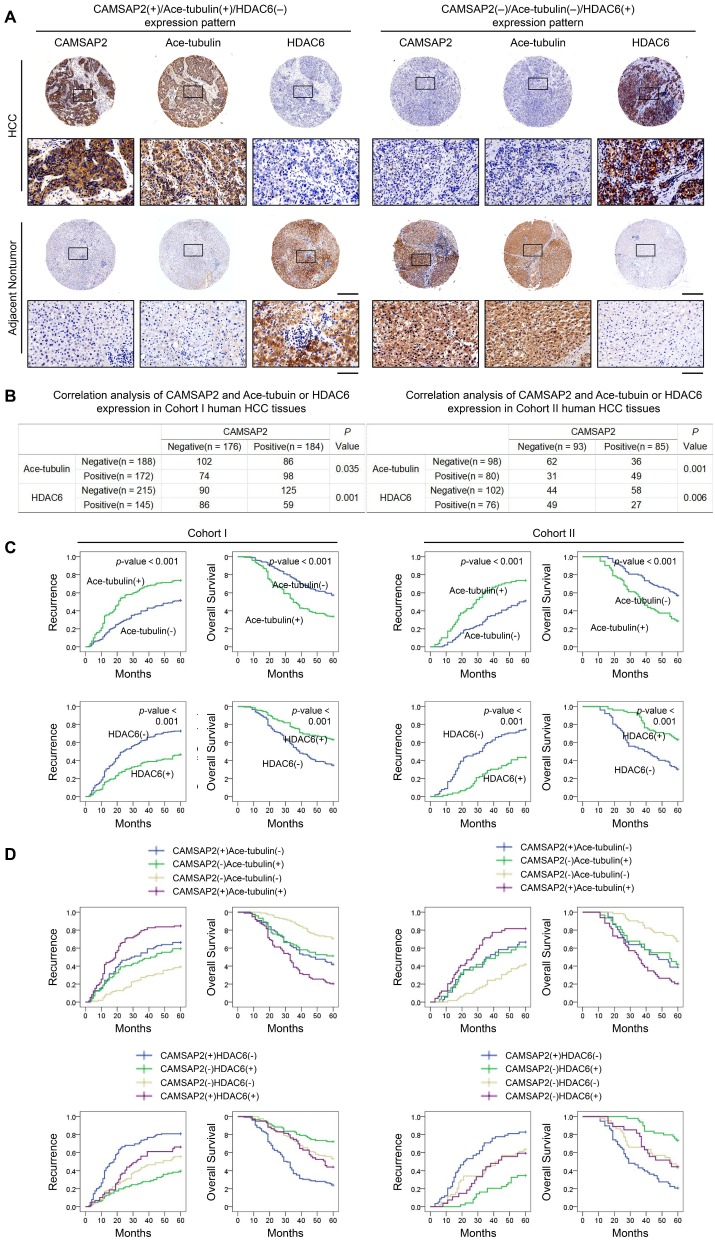
** Prognostic significance of the correlation between CAMSAP2 and acetylated α-tubulin or HDAC6 expression in HCC tissues.** (A) Representative IHC images of CAMSAP2, Ace-tubulin, and HDAC6 expression in HCC and adjacent nontumorous tissues. Scale bars, 500 µm (upper), 100 µm (lower). (B) Relationship between CAMSAP2 expression and Ace-tubulin or HDAC6 expression in two independent cohorts. (C) Kaplan-Meier analysis was used to determine the correlation between Ace-tubulin or HDAC6 expression and recurrence or OS in the two cohorts. (D) Kaplan-Meier analysis of the correlation of CAMSAP2/Ace-tubulin or CAMSAP2/HDAC6 coexpression with recurrence and OS in the two cohorts.

We evaluated the putative relationship between CAMSAP2 and acetylated α-tubulin or HDAC6 expression in the two independent HCC cohorts. IHC revealed that CAMSAP2 overexpression was positively correlated with acetylated α-tubulin, but inversely correlated with HDAC6 expression (Figure 7A-B). Positive and negative controls are shown in Figure S3B. Both western blotting and IF further confirmed that HDAC6 dramatically down-regulated in HCC tissues (Figure S6E-F). Surprisingly, unlike acetylated microtubules, detyrosinated α-tubulin scarcely is expressed in HCC tissue but is expressed specifically in endothelial tissue (Figure S5D). Further analysis of the clinicopathological characteristics in paired HCC and adjacent nontumor tissues indicated that upregulation of microtubule acetylation or downregulation of HDAC6 was significantly correlated with malignant tumor progression (Tables S6 and S7) and poor prognosis (Figure 7C). Kaplan-Meier analysis revealed that in both cohorts, patients with CAMSAP2(+)/acetylated α-tubulin(+) and CAMSAP2(+)/HDAC6(-) expression patterns showed the highest recurrence rate and lowest OS (Figure 7D).

### CAMSAP2 activates c-Jun to induce transrepression of HDAC6 through Trio-dependent activation of the Rac1/JNK pathway

**Figure 8:**
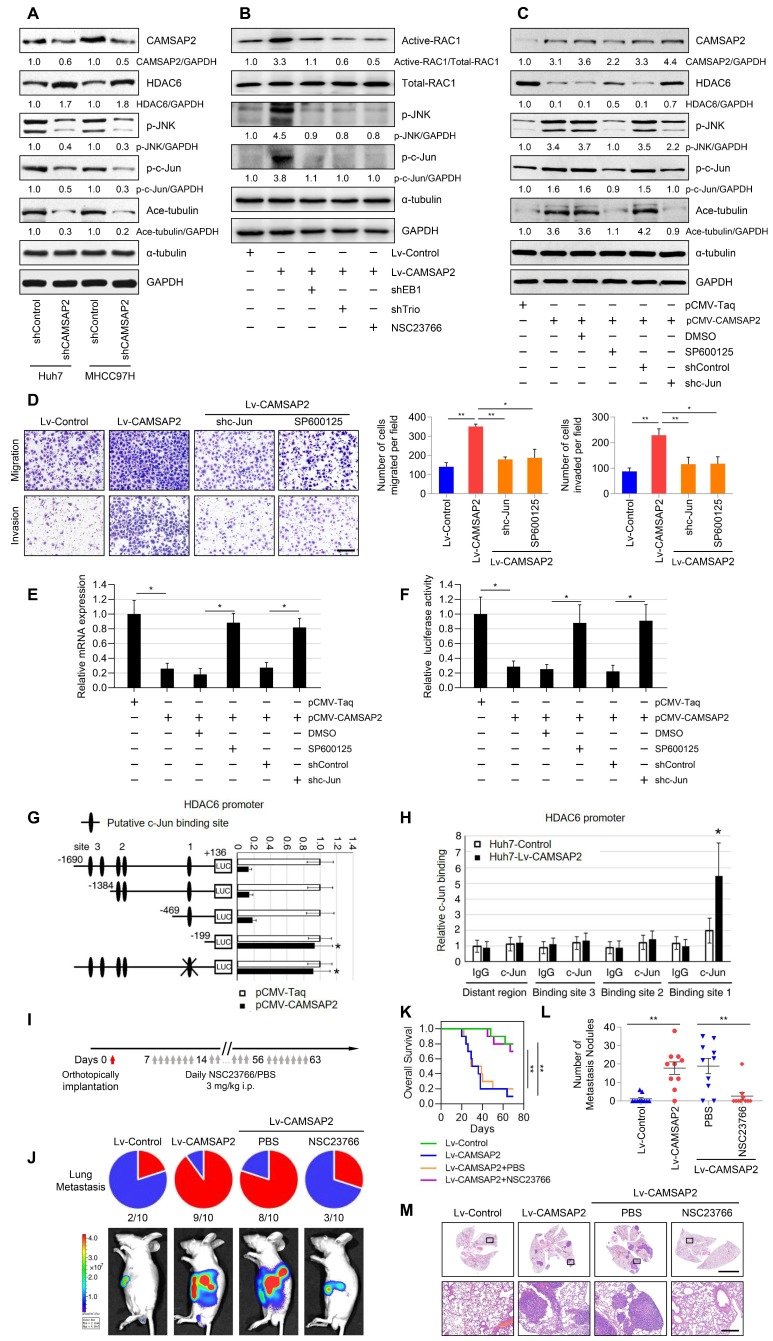
** CAMSAP2 activates c-Jun to induce transrepression of HDAC6 through Trio-dependent activation of Rac1/JNK pathway.** (A) Western blot analysis of CAMSAP2, HDAC6, Ace-tubulin, phosphorylated JNK, and c-Jun in control and CAMSAP2-depleted HCC cells. (B) Western blot analysis of active-Rac1, total Rac1, phosphorylated JNK, and c-Jun in the indicated HCC cells. NSC23766, a specific inhibitor was used to inactivate Rac1. (C) Western blot analysis of CAMSAP2, HDAC6, Ace-tubulin, phosphorylated JNK, and c-Jun in pCMV-CAMSAP2 and control cells (denoted as “pCMV-Taq”) treated with SP600125 or c-Jun knockdown lentivirus (denoted as “shc-Jun”). (D) Transwell assays of the indicated HCC cells. Migrating and invading cells were quantified in the right panel. Data are the mean ± SEM from triplicate experiments. Scale bar, 400 μm. **P < 0.01, *P < 0.05 (E) *HDAC6* mRNA levels in the indicated HCC cells as measured by RT-qPCR. Data are the mean ± SEM from triplicate experiments. *P < 0.05. (F) *HDAC6* promoter activity in the indicated HCC cells as measured by luciferase reporter assay. Data are the mean ± SEM from triplicate experiments. *P < 0.05. (G) Relative luciferase activities were measured after serially truncated and mutated *HDAC6* promoter constructs were cotransfected with pCMV-CAMSAP2 plasmid. (H) Chromatin immunoprecipitation assay demonstrating the direct binding of c-Jun to the *HDAC6* promoter in HCC cells. (I) Summary of the orthotopic metastatic model. (J) Incidence of lung metastasis and bioluminescence imaging of each group at 10 weeks after orthotopic xenografting with the indicated HCC cells. (K) OS of mice in the different groups. (L) Number of metastatic lung nodules observed in each group. *P < 0.05, **P < 0.01. (M) Representative H&E staining of lung tissues from each group. Scale bars, 500 mm (upper), 500 µm (lower).

Recent research findings have revealed that the HDAC6 expression is inhibited by c-Jun 21, a downstream transcription factor in Rac1/JNK signaling 22. Thus, we hypothesized that endogenous HDAC6 expression may be suppressed by CAMSAP2 through Trio-dependent activation of the Rac1/JNK/c-Jun pathway. CAMSAP2 knockdown decreased JNK and c-Jun phosphorylation and microtubule acetylation, whereas it increased HDAC6 protein expression in HCC cells (Figure 8A). Suppression of Rac1 activation upon treatment with the specific inhibitor NSC23766 or EB1 and Trio depletion abrogated JNK/c-Jun cascade activation induced by CAMSAP2 overexpression (Figure 8B). Pretreatment with the specific JNK inhibitor SP600125 or c-Jun knockdown to inactivate c-Jun in CAMSAP2-overexpressing Huh7 cells significantly rescued the decrease in HDAC6 expression induced by CAMSAP2 overexpression; α-tubulin acetylation was markedly decreased compared with the level in the controls (Figure 8C). Further, the elevated migration and invasion due to CAMSAP2 overexpression were significantly abrogated after SP600125 treatment or c-Jun knockdown in Huh7 cells (Figure 8D). The similar results also confirmed in MHCC97H cells (Figure S8A-B).

As activated c-Jun transrepresses target genes by directly interacting with their promoters, we examined whether JNK/c-Jun inactivates HDAC6 via a similar mechanism. RT-qPCR and luciferase reporter assays revealed that overexpression of CAMSAP2 inhibited *HDAC6* mRNA expression and promoter activity (Figure 8E-F). Pretreatment with SP600125 or c-Jun knockdown significantly rescued the decreases in *HDAC6* expression and promoter activity induced by CAMSAP2 overexpression (Figure 8E-F). Sequence analysis revealed three putative c-Jun-binding sites in the *HDAC6* promoter. Serial truncation experiments indicated that the first c-Jun-binding sites are critical for c-Jun-induced *HDAC6* transrepression (Figure 8G). A luciferase reporter assay revealed that mutation of the first c-Jun-binding site significantly ameliorated c-Jun-induced transrepression of the *HDAC6* promoter (Figure 8G). Chromatin immunoprecipitation assay results confirmed that c-Jun binds directly to the *HDAC6* promoter in HCC cells (Figure 8H). These data suggested that CAMSAP2 activates c-Jun to induce transrepression of *HDAC6* through Trio-dependent activation of the Rac1/JNK pathway.

### The Rac inhibitor NSC23766 suppresses CAMSAP2-mediated HCC invasion and metastasis

NSC23766, a Rac-specific inhibitor that does not affect Cdc42 or RhoA, has shown remarkable antitumor effects in multiple cancers 23-25. Therefore, we determined whether treatment with NSC23766 could reverse CAMSAP2-mediated HCC invasion and metastasis. NSC23766 effectively ameliorated the increase in GTP-Rac1 expression in Huh7-Lv-CAMSAP2 cells (Figure 4E) and abrogated JNK-c-Jun pathway activation induced by CAMSAP2 overexpression (Figure 8B). The elevated migration and invasion of CAMSAP2-overexpressing HCC cells were significantly abrogated after NSC23766 treatment (Figure 4F). We next evaluated an intrahepatic tumor implantation mouse model. Daily administration of NSC23766 in a single intraperitoneal dose of 3 mg/kg significantly reduced lung metastasis and prolonged the OS time in Huh7-Lv-CAMSAP2 cells (Figure 8I-K). H&E staining confirmed that the incidence of lung metastasis and the number of metastatic pulmonary nodules were significantly decreased upon NSC23766 treatment (Figure 8L-M). These findings suggested that NSC23766 inhibits CAMSAP2-mediated HCC invasion and metastasis by interrupting Rac1/JNK/c-Jun signaling and may provide a novel therapeutic option for HCC.

## Discussion

This study revealed that CAMSAP2, a microtubule minus-end-targeting protein, functions as an oncoprotein in HCC metastasis. CAMSAP2 upregulation promoted HCC cell invasion and metastasis *in vitro* and *in vivo*, whereas loss of CAMSAP2 suppressed HCC cell growth. Upregulated CAMSAP2 expression in HCC tissues was closely correlated with poor clinicopathological characteristics and prognosis, and clinical evidenced indicated that CAMSAP2 may serve as a prognostic factor for HCC metastasis. Moreover, the increased mRNA expression of CAMSAP2 was also observed in PAAD, STAD and COAD tissues based on TCGA data. Kaplan-Meier analysis revealed that PAAD, STAD and COAD patients with high levels of CAMSAP2 mRNA had a shorter OS and DFS. IHC staining confirmed that CAMSAP2 was upregulated in PAAD, STAD and COAD cancer specimens. The decreased migration and invasion capabilities also confirmed in SW1990 and SW620 CAMSAP2 knockdown cells. These results suggest that CAMSAP2 may play a carcinogenic role in a variety of tumors.

Our data suggested that CAMSAP2-mediated noncentrosomal microtubule rearrangement is essential for directional cell migration in HCC. Upon CAMSAP2 depletion, the partially noncentrosomal microtubule array was transformed into a completely radial centrosomal pattern and directional cell migration was impaired and characterized by defective Golgi reorienting towards the wound edge. CAMSAP2-decorated microtubule stretches function during interphase, but disappear during mitosis 5,10; however, we found that CAMSAP2, along with α-tubulin, exhibited a specific distribution pattern in HCC cells during mitosis. The molecular effects and mechanism of CAMSAP2 in mitosis remain to be further investigated (Figure S9A-B).

Microtubules are polarized cytoskeletal filaments with two structurally and functionally distinct ends. It is generally accepted that the plus end is the dynamic end, which recruits multiple +TIPs to couple the microtubule dynamics to specific cellular events 7. We investigated the regulatory roles of CAMSAP2 in microtubule dynamics by monitoring the behavior of EB1, which is considered to be the core of the +TIPs network as it autonomously tracks the growing microtubule plus ends 7, 18. Loss of CAMSAP2 led to reductions in the amount of polymerized a-tubulin as well as growing microtubule plus ends. Further, microtubule plus ends function as concentration devices for signaling molecules—Rho GTPase GEFs and kinases 6,19. We found that CAMSAP2 cooperates with the EB1/Trio complex to direct microtubule dynamics to invasive cell migration by promoting Rac1 signaling.

PTMs are emerging as important regulators of the microtubule cytoskeleton. Tubulin PTMs provide a mechanism for microtubules to adapt to specific cellular functions by regulating microtubule properties 13,14. α-Tubulin acetylated at lysine 40 is a hallmark of poor prognosis in patients with breast, head-and-neck, and pancreatic cancer 26-28. Acetylated microtubules accumulate toward the leading edge and regulate cell polarization, migration, and invasion 16,17. Therefore, we questioned whether CAMSAP2-mediated noncentrosomal microtubules are acetylated to promote HCC cell invasive migration. CAMSAP2 depletion decreased microtubule acetylation, whereas CAMSAP2 overexpression increased α-tubulin acetylation in HCC cells. The acetylated microtubule network, which typically extends protrusions towards the direction of migration, was disrupted in CAMSAP2-knockdown cells. However, there were no obvious changes in the expression of detyrosinated or tyrosinated α-tubulin after downregulation of CAMSAP2. Further, suppression of tubulin detyrosination by treatment with the inhibitor parthenolide had no significant effect on HCC cell migration and invasion. These results suggested that microtubule detyrosination may not be involved in HCC cells migration and invasion induced by CAMSAP2. Surprisingly, unlike acetylated microtubules, detyrosinated α-tubulin scarcely is expressed in HCC tissue but is expressed specifically in endothelial tissue, which suggests that detyrosinated microtubules may be related to angiogenesis in HCC.

Acetylation of α-tubulin at lysine 40 is tightly controlled by multiple acetyltransferases and deacetylases. Numerous enzymes, including but not limited to α-TAT1, ELP3, ARD1-NAT1, and GCN5, acetylate tubulin 29. In contrast, HDAC6 and sirtuin 2 are the only α-tubulin deacetylases described. Here, we identified HDAC6 as the predominant α-tubulin deacetylase in HCC cells: HDAC6 inhibition or depletion enhanced microtubule acetylation and promoted migration and invasion in HCC cells, whereas the protein level and distribution of acetylated microtubules were not obviously affected by sirtuin 2 suppression. We found that knockdown of CAMSAP2 in MHCC97H cells significantly increased HDAC6 expression, whereas HDAC6 was markedly downregulated in Huh7-Lv-CAMSAP2 cells with no apparent change in the expression of αTAT1 in the transfected HCC cells. Upregulation of HDAC6 abrogated the increases in migration and invasion as well as the expression and morphological alteration of acetylated α-tubulin induced by CAMSAP2 overexpression, suggesting that CAMSAP2 promotes microtubule acetylation to control invasive cell migration via an inhibitory interaction with HDAC6.

Notably, there are conflicting studies showing that HDAC6 has been implicated as an oncogene in several human cancers, including HCC 30, 31. However, recent studies from two other groups and our study showed that HDAC6 was uniquely down-regulated and endowed with tumor suppressive activity in HCC 32, 33. HDACs are widely considered to be critical epigenetic regulators involved in the regulation of malignancy and tumor microenvironment due to the importance of histone modifications 34. Unlike other HDACs, HDAC6 contains two catalytic domains and a conserved nuclear export signal, and thus may function predominately in the cytoplasm where it associates with modifications of nonhistones, such as α-tubulin 33, 35. Therefore, to better clarify the role and mechanism of HDAC6 in HCC, it would be too much of demand to use HDAC6 knockout cell lines in transplantation experiments or construct liver-specific HDAC6 conditional knockout mice.

Recent research findings have revealed that the HDAC6 expression is inhibited by c-Jun 21, a downstream transcription factor in Rac1/JNK signaling 22. Thus, we hypothesized that endogenous HDAC6 expression may be suppressed by CAMSAP2 through Trio-dependent activation of the Rac1/JNK/c-Jun pathway. Our results indicated that CAMSAP2 represses endogenous HDAC6 expression through Trio-dependent activation of the Rac1/JNK /c-Jun pathway. Ample experimental evidence indicated that HDAC6 is a direct transcriptional target of c-Jun.

In an attempt to develop a new pharmacological strategy against HCC metastasis based on our findings, we focused on inhibitors targeting Rac1 because CAMSAP2 inhibitors are currently not available. NSC23766 has shown remarkable antitumor effects in multiple cancers, including chronic myelogenous leukemia, glioblastoma, and colorectal cancer 23-25. Thus, we determined whether treatment with NSC23766 could reverse CAMSAP2-mediated HCC invasion and metastasis. We found that NSC23766 inhibited CAMSAP2-mediated HCC invasion and metastasis by interrupting Rac1/JNK/c-Jun signaling; thus, it may serve as a novel therapeutic option for HCC.

Thus, CAMSAP2 is functionally, mechanistically, and clinically oncogenic in HCC. Targeting CAMSAP2-mediated noncentrosomal microtubule acetylation may provide new therapeutic strategies for HCC metastasis (Figure S9C).

## Supplementary Material

Supplementary figures and tables.

## Abbreviations

CAMSAP: calmodulin‑regulated spectrin‑associated protein
COAD: colon adenocarcinoma
DFS: disease-free survival
GEFs: guanine nucleotide exchange factors
HCC: hepatocellular carcinoma
HDAC6: histone deacetylase 6
H&E: hematoxylin and eosin
IF: immunofluorescence
IHC: immunohistochemistry
K40R: lysine 40 to arginine
OS: overall survival
PAAD: pancreatic adenocarcinoma
PBS: phosphate-buffered saline
PCR: polymerase chain reaction
PTMs: post-translational modifications
siRNA: small interfering RNA
STAD: stomach adenocarcinoma
TCGA: The Cancer Genome Atlas dataset
+TIPs: plus-end-tracking proteins
Trio: triple functional domain protein
