# Supplementary material for: CAMSAP2-mediated noncentrosomal microtubule acetylation drives hepatocellular carcinoma metastasis
Source: Theranostics. 2020 Feb 19;10(8):3749–66. doi: 10.7150/thno.42596 (PMC7069094; doi:10.7150/thno.42596)
Supplement: Supplementary file 1 — Supplementary figures and tables. [file thnov10p3749s1.pdf]

Supplementary Materials

CAMSAP2-mediated noncentrosomal microtubule acetylation drives hepatocellular carcinoma metastasis

Dongxiao Li[1,2,#], Xiangming Ding[1,2,#], Meng Xie[1,2], Zheng Huang[1,2], Ping Han[1], Dean Tian[1,2], and Limin Xia[1,2*]

[1]Department of Gastroenterology, Tongji Hospital of Tongji Medical College, Huazhong University of Science and Technology, Wuhan 430030, Hubei Province, China

[2]Institute of Liver and Gastrointestinal Diseases, Tongji Hospital of Tongji Medical College, Huazhong University of Science and Technology, Wuhan 430030, Hubei Province, China

[#]These authors contributed equally to this work.

Materials and Methods

Patient samples and ethics statement

This study was approved by the Ethics Committee of Tongji Medical College of Huazhong University of Science and Technology and was performed in accordance with the ethical standards of the World Medical Association Declaration of Helsinki. Cohort I included 360 patients with HCC who underwent curative resection between

2005 and 2009 at the Tongji Hospital of Tongji Medical College (Wuhan, China). Cohort II included 178 patients with HCC who underwent curative resection between 2010 and 2012 at the Tongji Hospital of Tongji Medical College (Wuhan, China). Additionally, 90 pairs of fresh-frozen HCC and corresponding adjacent nontumor tissues and 20 pairs of fresh-frozen metastatic and matched primary HCC tissues were collected after curative resection at Tongji Hospital affiliated to Tongji Medical College (Wuhan, China) between 2015 and 2017.

Lentivirus construction and transfection

Lentiviruses encoding short hairpin (sh)RNAs were produced using pLKO.1-puro and pLKO.1-neo (Genechem, Shanghai, China and DesignGene, Shanghai, China) and were denoted as “shCAMSAP2,” “shEB1,” “shTrio,” “shHDAC6,” and “shc-Jun.” Recombinant lentivirus overexpressing CAMSAP2 was constructed using pLKO.1-puro (DesignGene Biotechnology, Shanghai, China) and was denoted as “Lv-CAMSAP2.” Cells were transfected using Lipofectamine 3000 (Invitrogen, CA, USA) per the manufacturer’s instructions. In brief, cells were transfected with lentivirus at a multiplicity of infection of 10–30 for 12 h. The medium was replaced with DMEM containing 10% FBS and the cells were cultivated for another 24 h. Transfected cells were selected with puromycin or G418 for 2 weeks. Target gene expression was confirmed by both western blotting and real-time reverse transcription (RT-q)PCR.

Plasmid construction

Plasmids were constructed as previously described [1,2]. All primers used are listed in Table S8. Briefly, a *HDAC6* promoter region (–1690/+136) was amplified from human genomic DNA using forward and reverse primers with *Mlu*I and *Xho*I sites at the 3’ and 5’ end, respectively. The amplification product was cloned into *Mlu*I- and *Xho*I-digested pGL3-Basic vector (Promega, Madison, WI). Constructs containing a deletion in the 5’-flanking region of the *HDAC6* promoter, (–1384/+136) HDAC6, (–469/+136) HDAC6, and (–199/+136) HDAC6, were constructed using the (–1690/+136) HDAC6 construct as the template. The QuikChange II Site-Directed Mutagenesis Kit (Stratagene, CA, USA) was utilized to mutate putative c-Jun binding sites in the *HDAC6* promoter region. The pSpCas9(BB)-2A-Puro (PX459, Addgene) vector that was used for the CRISPR/Cas9 knockout and the targeting sequence of 20 nucleotides was cloned into the vector (Qijing Biological Technology, Wuhan China). The sequence of sgRNA was designed as previously described [3]. The vectors encoding the α-tubulin point mutations at lysine 40 to arginine (K40R) was generated with the QuikChange II Site-Directed Mutagenesis Kit (DesignGene Biotechnology, Shanghai, China). Sequence integrity was verified by DNA sequencing (Qijing Biological Technology, Wuhan China).

***In-vivo* metastasis assay and bioluminescence imaging**

All experiments involving animals were approved by the experimental animal ethics committee of Tongji Medical College of Huazhong University of Science and

Technology. All animal procedures were carried out in accordance with the Guide for the Care and Use of Laboratory Animals and standards articulated in the Animal Research: Reporting of *In Vivo* Experiments. A metastatic HCC model was established in mice as previously described [1,2], with slight modifications. Briefly, 6×10^6 cells were suspended in PBS, mixed with Matrigel (BD Biosciences, CA, USA), and injected orthotopically into the left liver lobes of BALB/c nude mice (male, 4-week-old). Each treatment group consisted of 10 mice. For *in-vivo* monitoring, cells were infected with luciferase-expressing lentivirus (Lv-luc-blast, Hanbio, Shanghai, China) and selected with blasticidin for two weeks. D-Luciferin (Gold Biotechnology, USA) was injected weekly intraperitoneally into each mouse for monitoring tumor formation and metastasis, and images were captured with a Lago X optical imaging system (SI Imaging, USA). Lung tissues were dissected after 10 weeks, fixed with 4% paraformaldehyde, and stained with hematoxylin and eosin.

***In-vitro* migration and invasion assays**

Transwell assays were conducted as previously described [1,2]. The 3D Culture Hydrogel Kit (BeaverNano™, China) was used per the manufacturer's instructions [4]. Briefly, cells were resuspended in 120 μl of a 10% sucrose solution. After quickly mixing with an equal volume of 20% hydrogel solution, the mixture was immediately spread on a glass-bottom cell-culture dish (NEST, China). Cells were cultured for two weeks and imaged using an Olympus laser-scanning confocal microscope.

Microtubule fractionation assay

Microtubule fractionation assays were performed as described previously [5], with slight modifications. Cells were washed with PBS at 37 ℃ and incubated with microtubule-stabilizing buffer (100 mM PIPES, pH 6.8, 2 mM EGTA, 1 mM $MgCl_2$) supplemented with protease inhibitor cocktail and 0.5% NP-40 at 37 ℃ for 15 min. Lysates were centrifuged at 1,000×*g* for 10 min. The pelleted cells were lysed with sodium dodecyl sulfate (SDS) lysis buffer. The cell pellet and supernatant were mixed with sample buffer, boiled, and subjected to SDS-polyacrylamide gel electrophoresis (PAGE).

Microtubule repolymerization assay

Cells were treated with 15 μM nocodazole (HY-13520, MedChemExpress) at 4 ℃ for 30 min to completely depolymerize microtubules, incubated at 37 ℃ for 10 min after drug washout, fixed, and immunostained for α-tubulin (red) and γ-tubulin (green) to visualize microtubules repolymerization. Antibodies used are listed in Table S9.

GTPase activation assay

Rac1 activation was analyzed using a Rac1 Pulldown Activation Assay Kit (Cytoskeleton, Denver, USA) according to the manufacturer’s instructions.

Luciferase reporter assay

The Dual-Luciferase Reporter Assay System (Promega, Madison, WI) was utilized

per the manufacturer’s protocol. Briefly, cells transfected with the indicated plasmids were lysed; the lysates were centrifuged at maximum speed for 1 min. Luciferase activity was measured using a TD20/20 Luminometer (Turner Biosystems, USA) and was normalized to Renilla luciferase activity.

Co-immunoprecipitation assay

Co-immunoprecipitation was performed as described previously [6]. Briefly, cells were lysed on ice with lysis buffer containing 1% NP-40 (Promoter, China) for 30 min. The lysates were centrifuged at 12,000×*g* for 15 min. The supernatants were incubated with Protein G-conjugated Sepharose beads (Santa Cruz Biotechnology, TX, USA) and the appropriate antibodies at 4 ℃ overnight. Immunoprecipitates were washed thrice with lysis buffer and separated by SDS-PAGE. The antibodies used are listed in Table S9.

Chromatin immunoprecipitation assay

Chromatin immunoprecipitation was carried out as described previously [7]. Briefly, transfected cells were cross-linked in 1% formaldehyde at 37 ℃ for 10 min. After washing with PBS, the cells were resuspended in 300 μl of lysis buffer and sonicated to fragment the DNA. A slurry of Protein G-Sepharose and herring sperm DNA (Sigma-Aldrich, USA) was used to clear the supernatant. The cleared supernatant was incubated with specific antibodies or an isotype control IgG in the presence of Protein G-Sepharose beads and herring sperm DNA for 2 h. The antibodies used are listed in

Table S9. The DNA was removed from the beads by immersion in a 1.1 M $NaHCO_3$ and 1% SDS solution at 65 ℃ for 6 h and purified using a QIAQuick PCR Purification Kit (Qiagen, USA). The primers used are listed in Table S8.

Quantitative reverse-transcription (RT-q)PCR

Total RNA was extracted using TRIzol Reagent (TaKaRa, Otsu, Japan) and reverse-transcribed using the PrimeScript RT Reagent Kit (TaKaRa) per the manufacturer's instructions. qPCRs were run using SYBR Premix ExTaq (TaKaRa, Otsu, Japan) on ABI StepOne system (Applied Biosystems, Carlsbad, CA, USA). The thermal cyclers were as follows: 40 cycles of 95 ℃ for 30 s, 95 ℃ for 5 s, and 60 ℃ for 30 s. The $2^{-\Delta\Delta Ct}$ method was used to determine fold differences between samples.

Western blotting

Western blot analyses were conducted as previously described [1,2]. Antibodies are listed in Table S9.

Tissue microarray analysis and immunohistochemistry (IHC)

Tissue microarrays were constructed as described previously [1,2]. For IHC, paraffin-embedded tissues were cut into 4-μm-thick sections. The sections were deparaffinized in dimethylbenzene, subjected to gradient alcohol dehydration, treated with 3% H_2O_2 to block endogenous peroxidase, and incubated with primary antibodies overnight. Then, the sections were incubated with the secondary antibody

at room temperature for 30 min. Immunoreactivity was visualized with diaminobenzidine and the sections were counterstained with hematoxylin. Antibodies are listed in Table S9.

IHC was evaluated by two independent observers who were blinded to the clinical and outcome data. The percentage of positive cells was scored on a scale of 0 to 4: 0 (negative), 1 (1%–25%), 2 (26%–50%), 3 (51%–75%), or 4 (76%–100%). The staining intensity was graded on a scale of 0 to 3: 0 (negative), 1 (weak), 2 (medium), or 3 (strong). Final immuno-activity scores were calculated by multiplying the above two scores, with final scores ranging from 0 to 12. Immuno-activity was considered positive if the final score was ≥4, or negative if it was <4.

Anchorage-dependent tumor growth assay

Eight-hundred cells were seeded into a 6-well cell-culture plate and cultivated in DMEM supplemented with 10% FBS at 37 ℃ in a humidified atmosphere containing 5% CO_2 for 14 days. After removing the medium and washing twice with PBS, the cells were fixed with 4% paraformaldehyde at room temperature for 15 min and stained with crystal violet for another 15 min. The plates were imaged using a camera.

Databases

mRNA expression data of CAMSAPs in liver cancer specimens compared to normal liver tissues were obtained from Cancer Genome Atlas (https://cancergenome.nih.gov).

IHC staining data of CAMSAPs in liver cancer tissues were downloaded from the Human Protein Atlas program. Kaplan–Meier analysis of The Cancer Genome Atlas data was conducted in cBioportal.

Statistical analysis

Data are reported as the mean ± SEM of triplicate experiments. Means were compared using Student's *t*-test. Categorical data were analyzed using Fisher's exact test. Kaplan–Meier analysis and log-rank tests were used to analyze the cumulative recurrence and survival rates. The Mann–Whitney U test was used for statistical quantitative analysis of IF signal intensity. The Cox proportional hazards model was used for univariate and multivariate analyses. All statistical analyses were conducted using SPSS (version 19.0). A value of $P < 0.05$ was considered significant.

References

1. Huang W, Chen Z, Zhang L, Tian D, Wang D, Fan D, et al. Interleukin-8 induces expression of FOXC1 to promote transactivation of CXCR1 and CCL2 in hepatocellular carcinoma cell lines and formation of metastases in mice. Gastroenterology. 2015; 149: 1053-1067 e14.
2. Huang W, Chen Z, Shang X, Tian D, Wang D, Wu K, et al. Sox12, a direct target of FoxQ1, promotes hepatocellular carcinoma metastasis through up-regulating Twist1 and FGFBP1. Hepatology. 2015; 61: 1920-1933.
3. Wu J, de Heus C, Liu Q, Bouchet BP, Noordstra I, Jiang K, et al. Molecular Pathway of Microtubule Organization at the Golgi Apparatus. Dev Cell. 2016; 39: 44-60.
4. Han P, Fu Y, Liu J, Wang Y, He J, Gong J, et al. Netrin-1 promotes cell

migration and invasion by down-regulation of BVES expression in human hepatocellular carcinoma. Am J Cancer Res. 2015; 5: 1396-1409.

5. Nagae S, Meng W, Takeichi M. Non-centrosomal microtubules regulate F-actin organization through the suppression of GEF-H1 activity. Genes Cells. 2013; 18: 387-396.

6. Ding X, Li D, Li M, Wang H, He Q, Wang Y, et al. SLC26A3 (DRA) prevents TNF-alpha-induced barrier dysfunction and dextran sulfate sodium-induced acute colitis. Lab Invest. 2018; 98: 462-476.

7. Xia L, Huang W, Bellani M, Seidman MM, Wu K, Fan D, et al. CHD4 has oncogenic functions in initiating and maintaining epigenetic suppression of multiple tumor suppressor genes. Cancer Cell. 2017; 31: 653-668 e7.

Figure legends

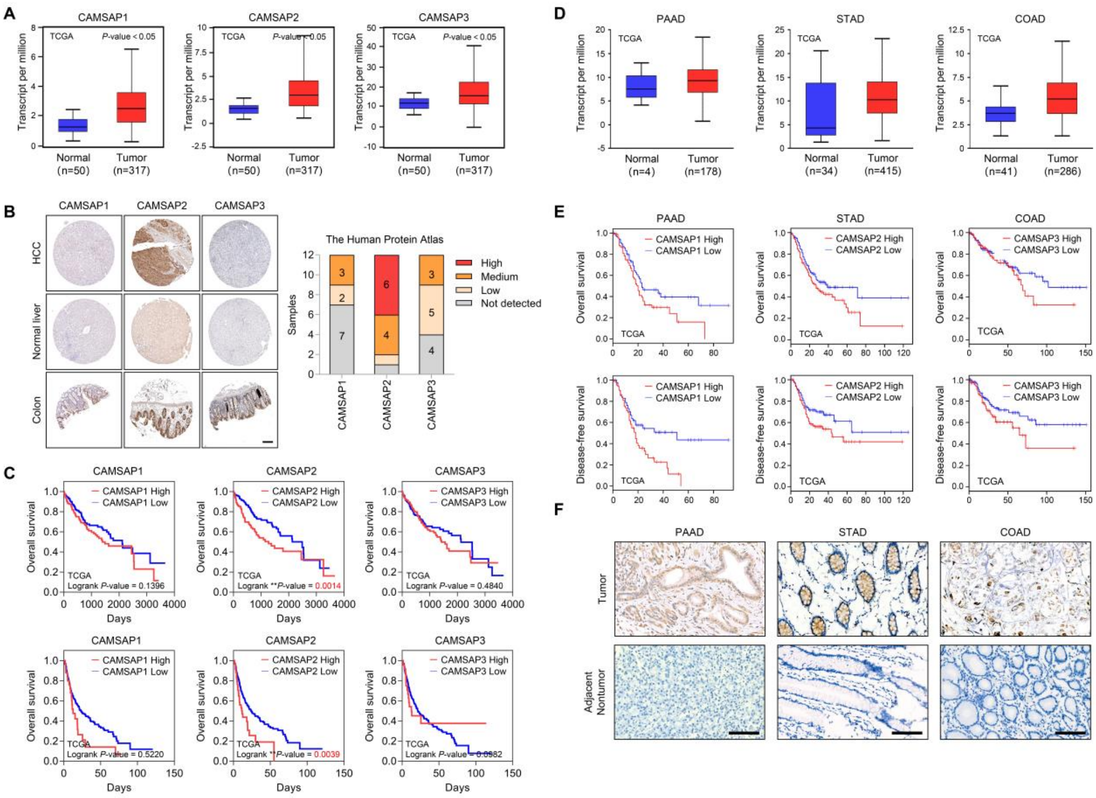

Figure S1. (A) Representative data obtained from The Cancer Genome Atlas dataset showing relative mRNA expression levels of CAMSAPs in normal liver versus liver cancer tissues. Box-and-whisker plots indicate the median (horizontal line), interquartile range (box), and 10th–90th percentiles (whiskers). *P < 0.05. (B) IHC staining levels for CAMSAPs in liver cancer tissues obtained from the Human Protein Atlas database. (C) Kaplan–Meier analysis of data obtained from The Cancer Genome Atlas database revealed a correlation between CAMSAP mRNA expression levels and overall and disease-free survival. (D) Representative data obtained from the Cancer Genome Atlas dataset showing relative mRNA expression levels of CAMSAP2 in PAAD, STAD and COAD tissues compared to the levels in normal tissues. Box-and-whisker plots indicate the median (horizontal line), interquartile range (box),

and 10th–90th percentiles (whiskers). (E) Kaplan–Meier analysis of data obtained from the Cancer Genome Atlas revealed a correlation between CAMSAP2 mRNA expression levels and overall and disease-free survival in PAAD, STAD and COAD. (F) IHC staining for CAMSAP2 in PAAD, STAD and COAD tissues and normal tissues. Scale bars, 100 μm.

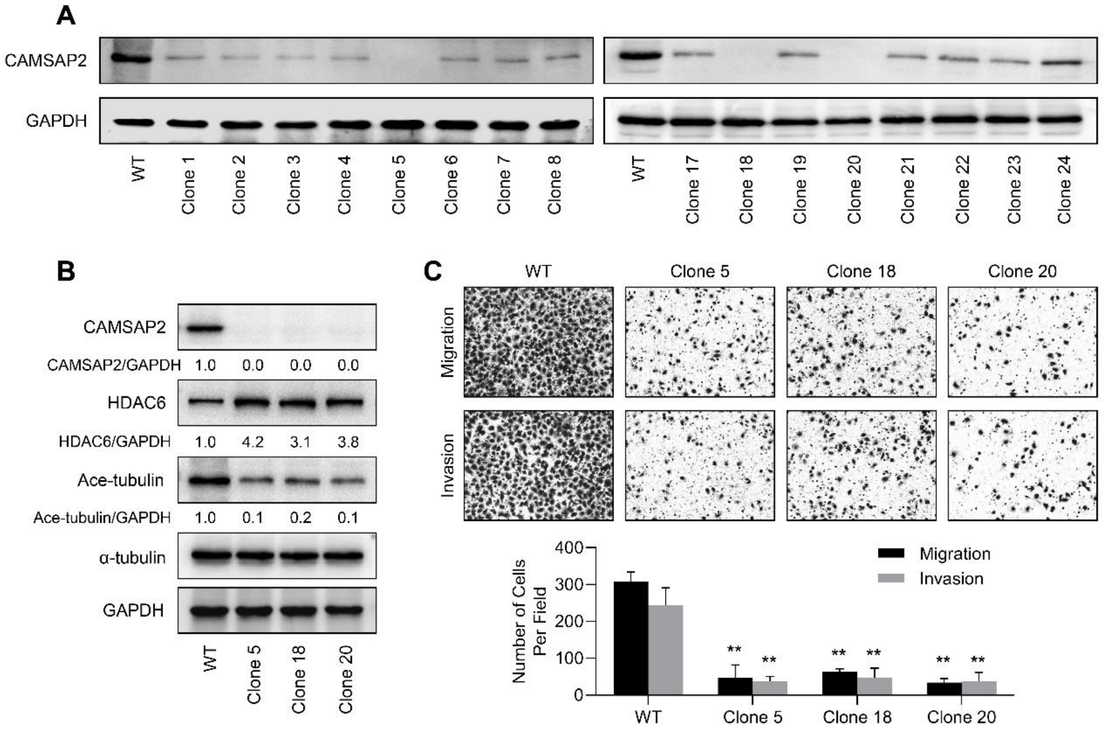

Figure S2. (A) Protein levels of CAMSAP2 in the indicated HCC cells as determined by Western blotting. (B) Western blot analysis of CAMSAP2, HDAC6 and Ace-tubulin in the indicated HCC cells. (C) Transwell assays of the indicated HCC cells. Migrating and invading cells were quantified in the lower panel. Data are the mean ± SEM from triplicate experiments. **P < 0.01. Scale bar, 400 μm.

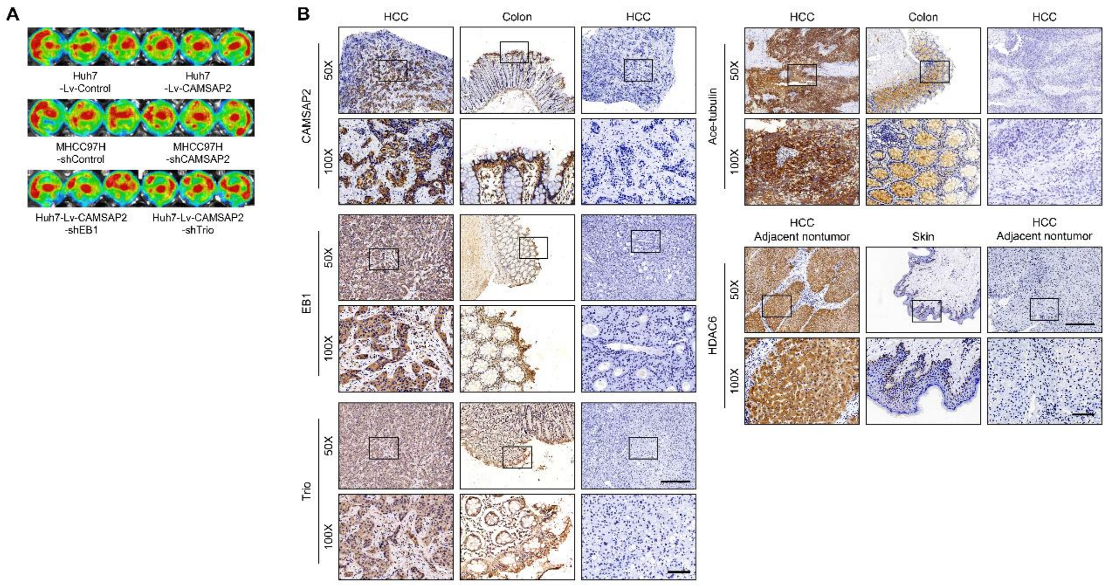

Figure S3. (A) Bioluminescence imaging of the indicated HCC cells before orthotopic implantation. (B) IHC staining of positive and negative controls of the indicated groups. Scale bars, 50 μm. Magnifications of the boxed areas are shown in the lower panels. Scale bars, 200 μm.

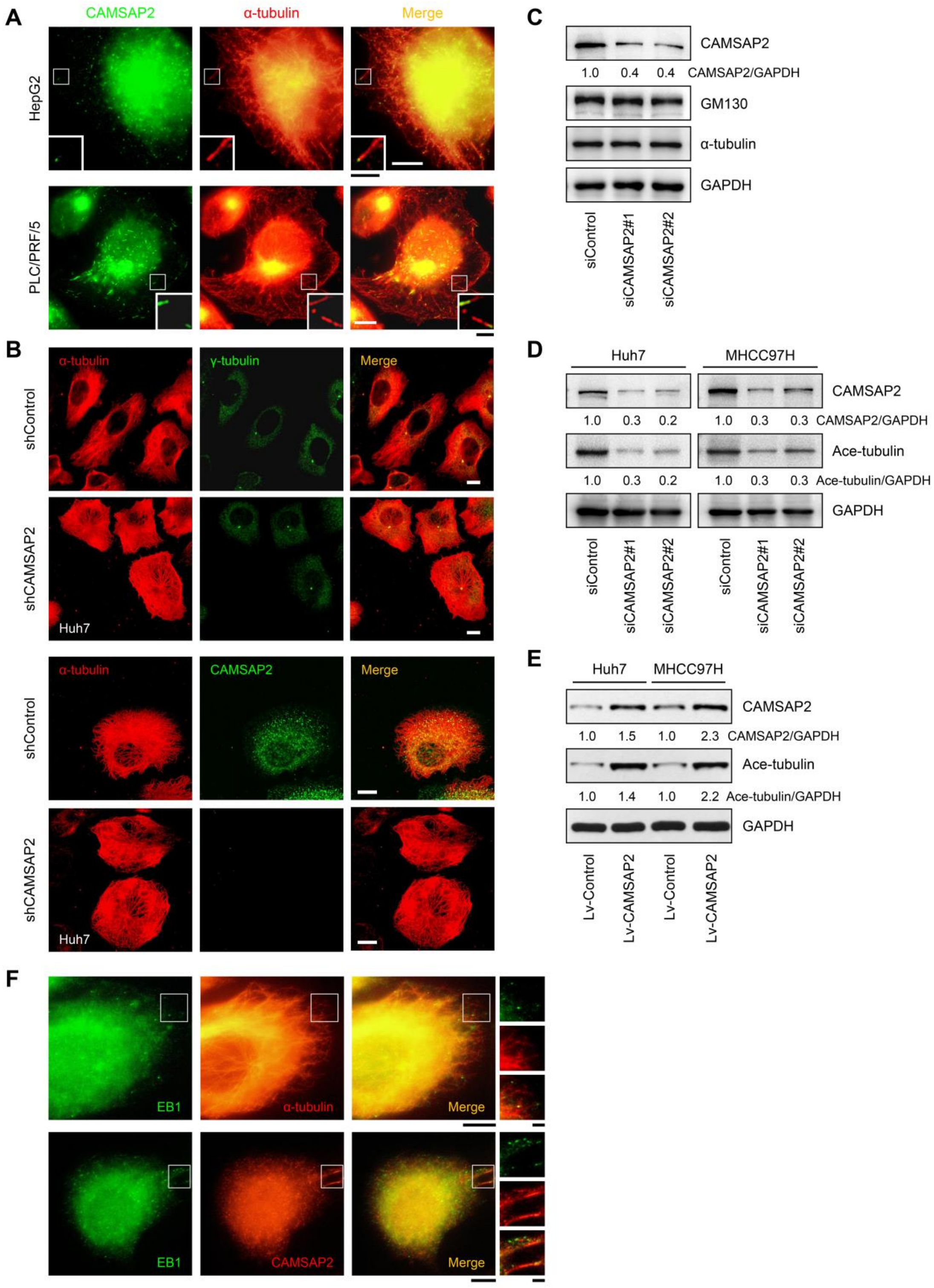

Figure S4. (A) Double staining for CAMSAP2 (green) and α-tubulin (red) in HepG2 and PLC/PRF/5 cells. Scale bars, 100 μm. Magnifications of the boxed areas are shown in the insets. Scale bars, 20 μm. (B) Double immunostaining for α-tubulin (red) and the centrosome marker γ-tubulin/CAMSAP2 (green) in control and

CAMSAP2-depleted Huh7 cells. Scale bars, 100 μm. (C) Western blot analysis of CAMSAP2 and GM130 in the indicated cells. (D, E) Western blot analysis of CAMSAP2 and Ace-tubulin in the indicated HCC cells. (F) IF staining of EB1 (green) and α-tubulin/CAMSAP2 (red) in MHCC97H cells. Scale bars, 100 μm, 20 μm (insert).

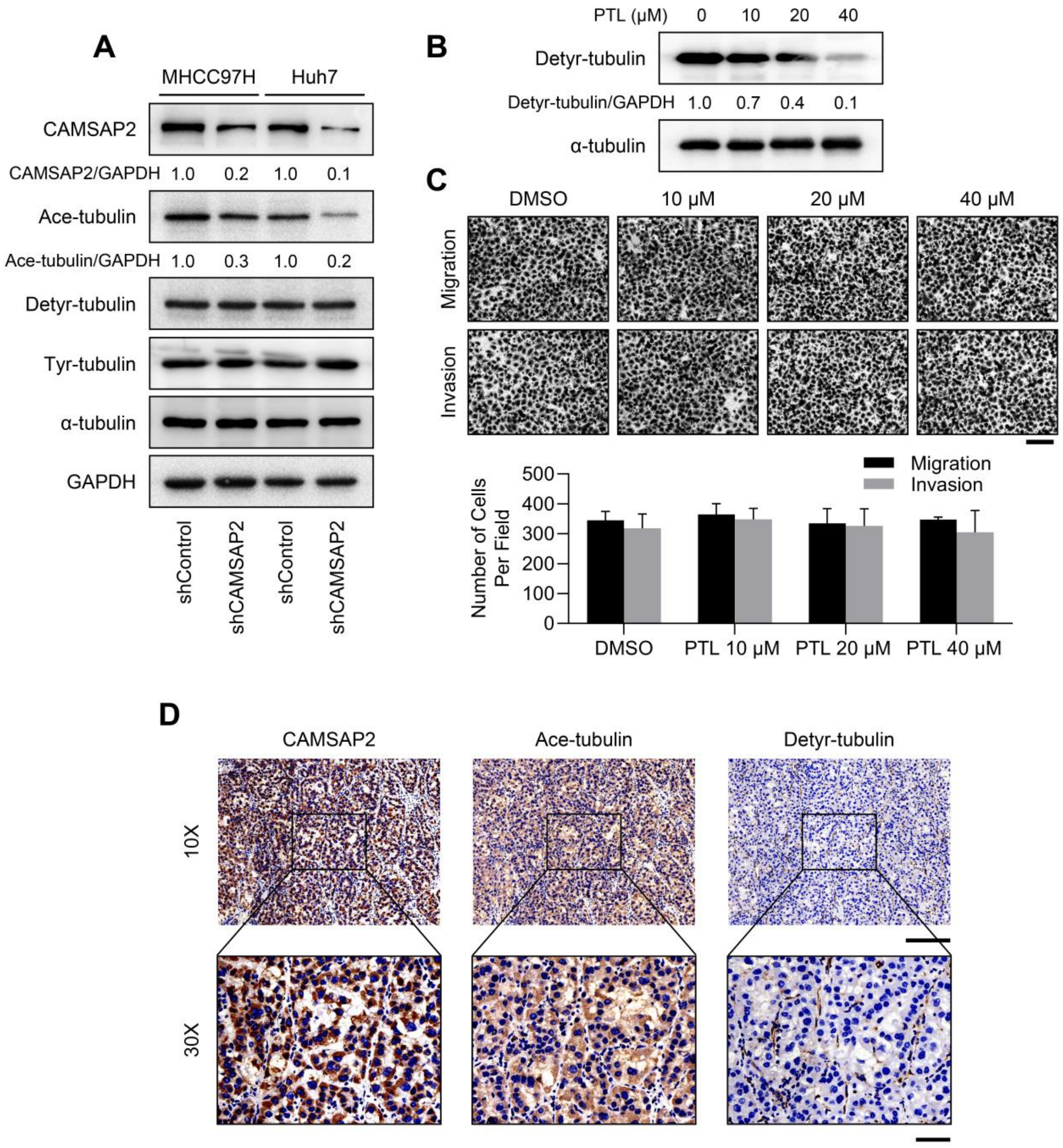

Figure S5. (A) Protein levels of CAMSAP2, Ace-tubulin, detyrosinated α-tubulin (Detyr-tubulin) and tyrosinated α-tubulin (Tyr-tubulin) in the indicated cells as

determined by Western blotting. (B) Western blot analysis of Detyr-tubulin in MHCC97H cells treated with parthenolide (PTL). (C) Transwell assays of the indicated HCC cells. Migrating and invading cells were quantified in the lower panel. Data are the mean ± SEM from triplicate experiments. Scale bar, 400 μm. (D) Representative IHC staining of CAMSAP2, Ace-tubulin, and Detyr-tubulin in HCC and corresponding adjacent nontumorous tissues. Scale bars, 50 μm. The enlargements of boxed regions are shown in the lower panels. Scale bars, 200 μm.

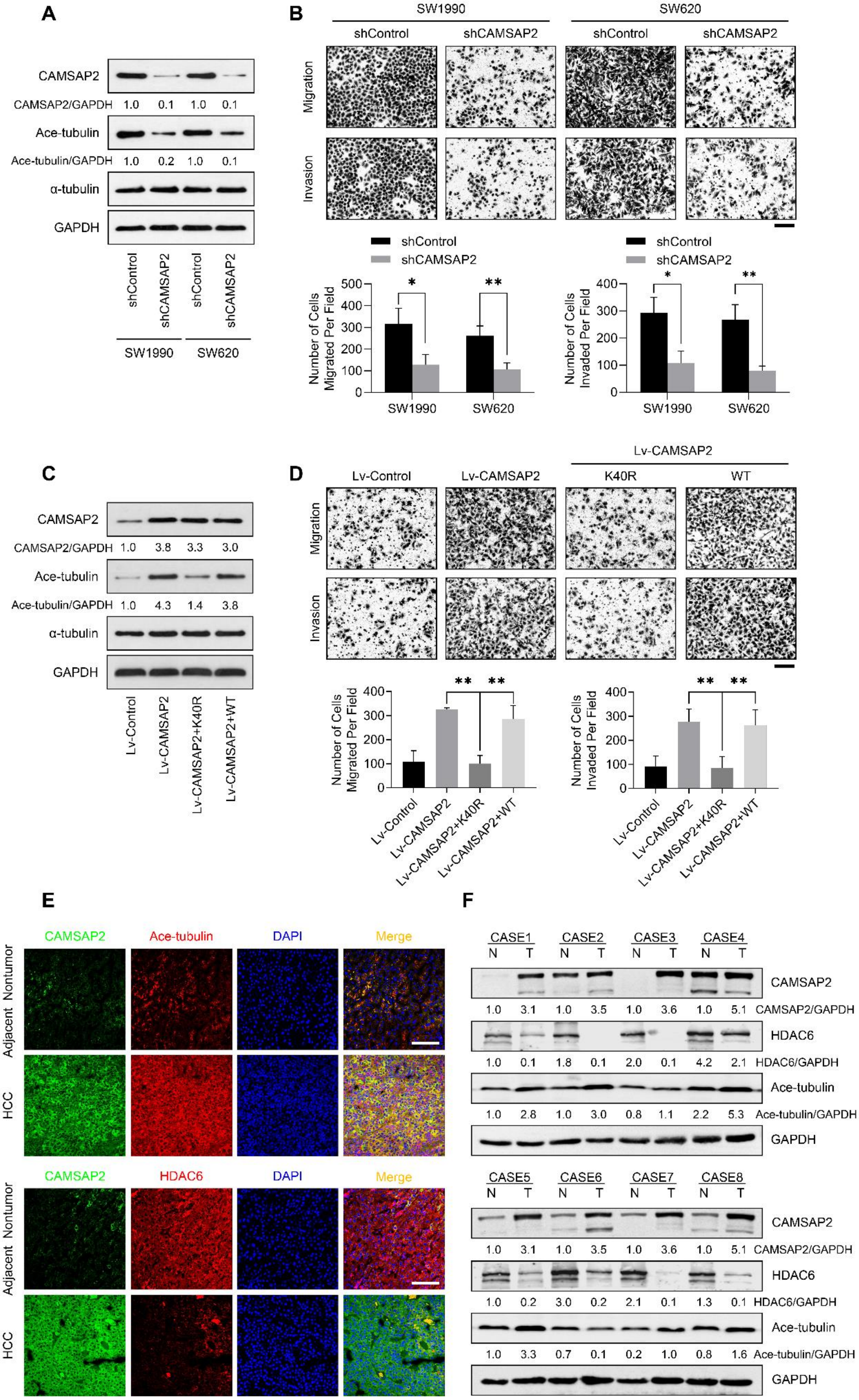
A
CAMSAP2
CAMSAP2/GAPDH 1.0 0.1 1.0 0.1
Ace-tubulin
Ace-tubulin/GAPDH 1.0 0.2 1.0 0.1
α-tubulin
GAPDH
shControl
shCAMSAP2
shControl
shCAMSAP2
SW1990
SW620
B
SW1990
SW620
shControl
shCAMSAP2
shControl
shCAMSAP2
Migration
Invasion
shControl
shCAMSAP2
Number of Cells Migrated Per Field
Number of Cells Invaded Per Field
SW1990
SW620
C
CAMSAP2
CAMSAP2/GAPDH 1.0 3.8 3.3 3.0
Ace-tubulin
Ace-tubulin/GAPDH 1.0 4.3 1.4 3.8
α-tubulin
GAPDH
Lv-Control
Lv-CAMSAP2
Lv-CAMSAP2+K40R
Lv-CAMSAP2+WT
D
Lv-Control
Lv-CAMSAP2
Lv-CAMSAP2
K40R
WT
Migration
Invasion
Number of Cells Migrated Per Field
Number of Cells Invaded Per Field
Lv-Control
Lv-CAMSAP2
Lv-CAMSAP2+K40R
Lv-CAMSAP2+WT
E
CAMSAP2
Ace-tubulin
DAPI
Merge
Adjacent Nontumor
HCC
CAMSAP2
HDAC6
DAPI
Merge
Adjacent Nontumor
HCC
F
CASE1 CASE2 CASE3 CASE4
N T N T N T N T
CAMSAP2
1.0 3.1 1.0 3.5 1.0 3.6 1.0 5.1 CAMSAP2/GAPDH
HDAC6
1.0 0.1 1.8 0.1 2.0 0.1 4.2 2.1 HDAC6/GAPDH
Ace-tubulin
1.0 2.8 1.0 3.0 0.8 1.1 2.2 5.3 Ace-tubulin/GAPDH
GAPDH
CASE5 CASE6 CASE7 CASE8
N T N T N T N T
CAMSAP2
1.0 3.1 1.0 3.5 1.0 3.6 1.0 5.1 CAMSAP2/GAPDH
HDAC6
1.0 0.2 3.0 0.2 2.1 0.1 1.3 0.1 HDAC6/GAPDH
Ace-tubulin
1.0 3.3 0.7 0.1 0.2 1.0 0.8 1.6 Ace-tubulin/GAPDH
GAPDH

Figure S6. (A) Protein levels of CAMSAP2 and Ace-tubulin in the indicated cells as determined by Western blotting. (B) Transwell assays of the indicated HCC cells. Migrating and invading cells were quantified in the lower panel. Data are the mean ± SEM from triplicate experiments. *$P < 0.05$, **$P < 0.01$. Scale bar, 400 μm. (C) Protein levels of CAMSAP2 and Ace-tubulin in the indicated cells as determined by Western blotting. (D) Transwell assays of the indicated HCC cells. Migrating and invading cells were quantified in the lower panel. Data are the mean ± SEM from triplicate experiments. **$P < 0.01$. Scale bar, 400 μm. (E) Representative IHC staining of CAMSAP2 (green) and Ace-tubulin/HDAC6 (red) in HCC and corresponding adjacent nontumorous tissues. Scale bars, 50 μm. (F) Protein expression of CAMSAP2, Ace-tubulin and HDAC6 in 8 paired HCC and adjacent nontumorous tissues was detected by Western blotting. N, adjacent nontumorous tissues; T, tumor tissues.

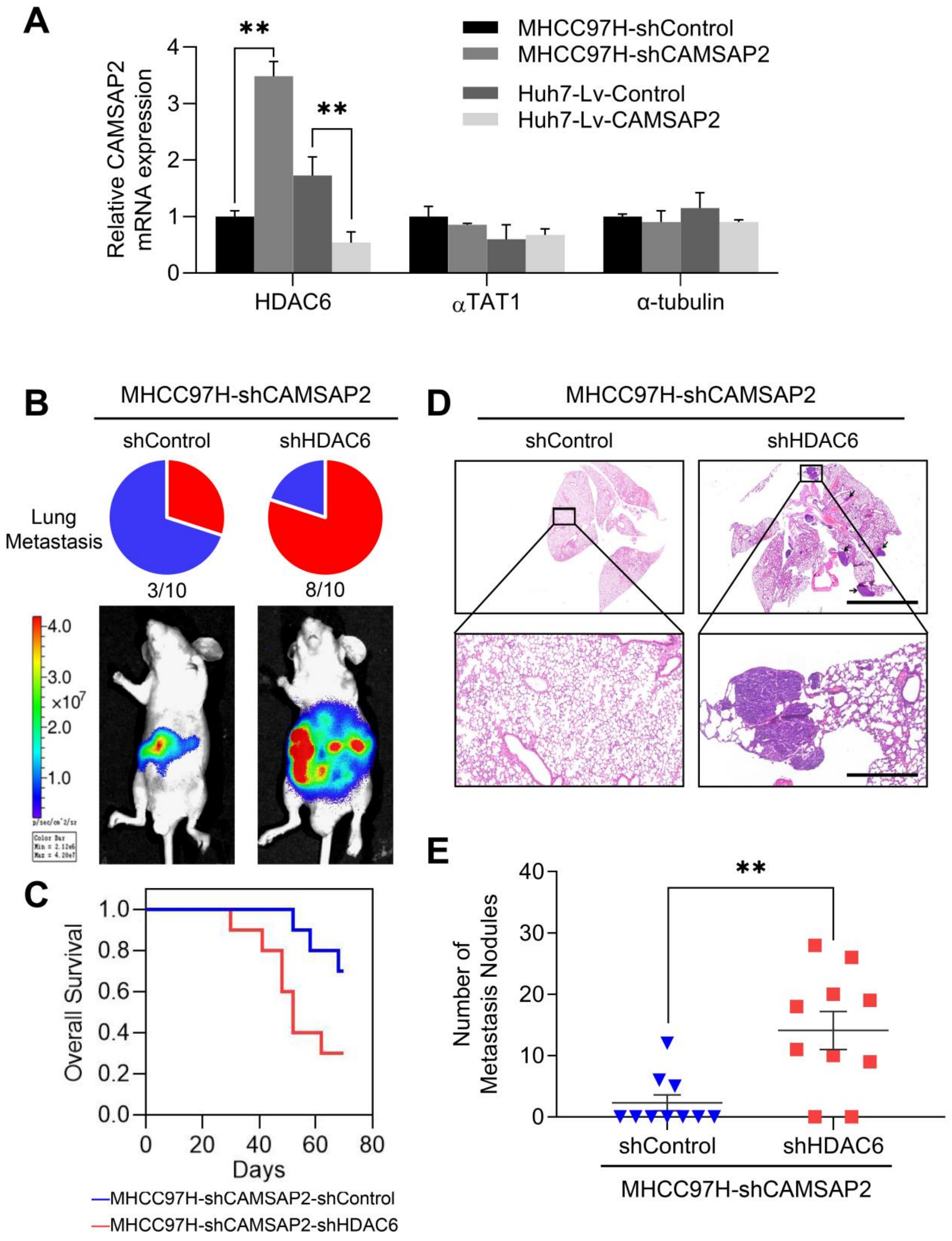

Figure S7. (A) HDAC6 and αTAT1 mRNA expression in the indicated HCC cells was measured by RT-qPCR. The data are presented as the mean ± SEM for triplicate experiments. **P < 0.01. (B) Incidence and of lung metastasis and bioluminescence imaging of each group at 10 weeks after orthotopic xenografting with the indicated

HCC cells. (C) Overall survival of mice in the different groups. (D) Representative H&E staining of lung tissues from each group. Scale bars, 500 mm (upper), 500 μm (lower). (E) Number of metastatic lung nodules observed in each group. **P < 0.01.

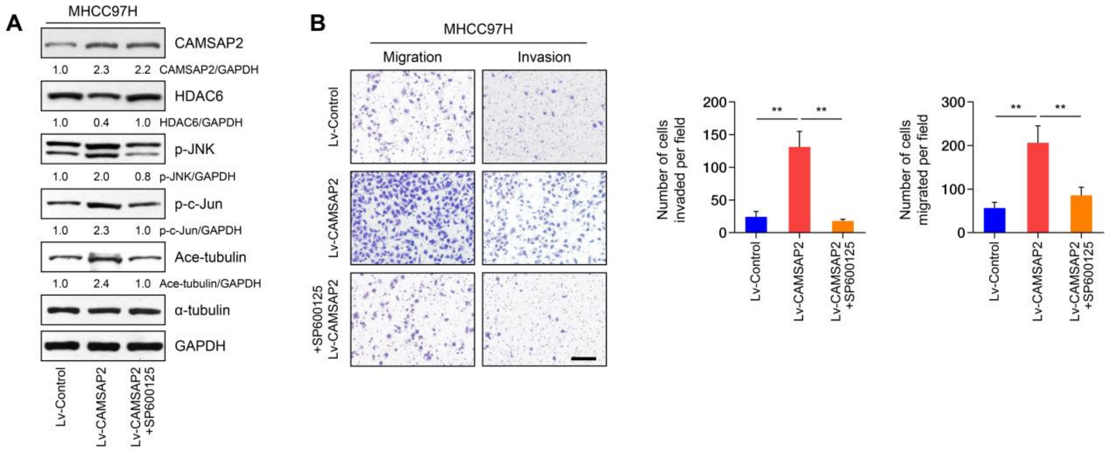

Figure S8. (A) Western blot analysis of CAMSAP2, HDAC6, Ace-tubulin, phosphorylated JNK, and c-Jun in the indicated cells. (B) Transwell assay of the indicated cells. Migrating and invading cells are quantified in the right panel. Data are the mean ± SEM from triplicate experiments. Scale bars, 50 μm. *P < 0.05, **P < 0.01.

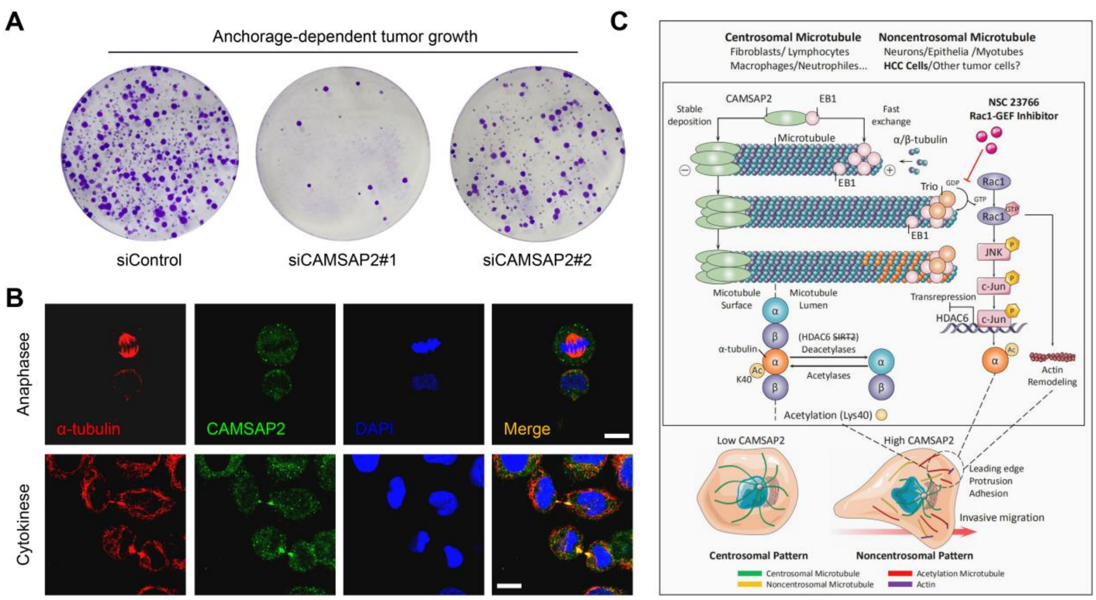

Figure S9. (A) Anchorage-dependent tumor growth assay of HCC cells treated with the indicated siRNA. (B) Localization of endogenous CAMSAP2 during anaphase and cytokinesis in MHCC97H cells. IF staining of CAMSAP2 (green), α-tubulin (red), and DNA (DAPI, blue). Scale bars, 100 μm. (C) Schematic diagram of the regulatory mechanism of CAMSAP2-mediated noncentrosomal microtubule acetylation driving HCC metastasis.

Table S1. Correlation between CAMSAP2 expression and clinicopathological characteristics of HCC in human HCC tissues from two independent cohorts

Clinicopathological variables		Cohort I Tumor CAMSAP2 expression Negative (n=176)	Cohort I Tumor CAMSAP2 expression Positive (n=184)	Cohort I P Value	Cohort II Tumor CAMSAP2 expression Negative (n=93)	Cohort II Tumor CAMSAP2 expression Positive (n=85)	Cohort II P Value
Age		52.49(9.09)	51.70(9.71)	0.425	51.37(11.95)	50.56(11.47)	0.649
Sex	female	26	30	0.772	19	15	0.705
	male	150	154		74	70	
Serum AFP	≤20ng/ml	33	32	0.785	21	18	0.858
	>20ng/ml	143	152		72	67	
Child-pugh score	Class A	143	153	0.680	67	58	0.624
	Class B	33	31		26	27	
Tumor number	single	127	110	0.015	60	39	0.016
	multiple	49	74		33	46	
Maximal tumor size	≤5cm	103	73	<0.001	45	27	0.032
	>5cm	73	111		48	58	
Tumor encapsulation	absent	33	76	<0.001	30	51	<0.001
	present	143	108		63	34	
Microvascular invasion	absent	119	85	<0.001	61	36	0.003
	present	57	99		32	49	
Tumor differentiation	I-II	147	122	<0.001	77	52	0.001
	III-IV	29	62		16	33	
TNM stage	I-II	154	118	<0.001	79	50	<0.001
	III	22	66		14	35	

Table S2. Uni- and multivariate analyses of factors associated with survival and recurrence of 360 HCCs (cohort I)

Variables	Recurrence						Survival					
	Univariate analysis			multivariate analysis			Univariate analysis			multivariate analysis		
	HR	95% CI	P value	HR	95% CI	*P* value	HR	95% CI	*P* value	HR	95% CI	*P* value
Age	0.990	0.976-1.004	0.156				0.989	0.974-1.004	0.142			
Sex (female versus male)	0.845	0.578-1.235	0.385				0.902	0.604-1.346	0.613			
Serum AFP (≤20 versus >20 ng/ml)	0.801	0.567-1.131	0.208				0.729	0.494-1.076	0.111			
Child-pugh score (A versus B)	1.074	0.755-1.529	0.690				0.974	0.673-1.411	0.890			
Tumor number (single versus multiple)	0.447	0.343-0.583	<0.001	0.839	0.596-1.182	0.316	0.443	0.334-0.589	<0.001	0.992	0.688-1.430	0.964
Maximal tumor size (≤5 versus >5 cm)	0.539	0.412-0.705	<0.001	0.942	0.694-1.280	0.703	0.535	0.401-0.715	<0.001	1.040	0.746-1.452	0.816
Tumor encapsulation (absent versus present)	2.274	1.733-2.984	<0.001	1.150	0.829-1.596	0.403	2.451	1.840-3.266	<0.001	1.183	0.833-1.680	0.349
Microvascular invasion (absent versus present)	0.420	0.323-0.548	<0.001	0.595	0.436-0.812	0.001	0.410	0.308-0.545	<0.001	0.647	0.462-0.907	0.011
Tumor differentiation (I-II versus III-IV)	0.304	0.230-0.402	<0.001	0.813	0.556-1.189	0.286	0.275	0.205-0.368	<0.001	0.700	0.476-1.029	0.070
TNM stage (I-II versus III)	0.187	0.141-0.249	<0.001	0.330	0.212-0.515	<0.001	0.172	0.127-0.232	<0.001	0.288	0.180-0.459	<0.001
**CAMSAP2** expression (negative versus positive)	0.428	0.326-0.562	<0.001	0.631	0.469-0.849	**0.002***	0.397	0.295-0.536	<0.001	0.631	0.456-0.875	**0.006***

Table S3. Uni- and multivariate analyses of factors associated with survival and recurrence of 178 HCCs (cohort II)

Variables	Recurrence						Survival					
	Univariate analysis			multivariate analysis			Univariate analysis			multivariate analysis		
	HR	95% CI	*P* value	HR	95% CI	*P* value	HR	95% CI	*P* value	HR	95% CI	*P* value
Age	0.988	0.973-1.004	0.153				0.983	0.967-0.999	0.043			
Sex (female versus male)	1.136	0.711-1.813	0.594				1.075	0.652-1.774	0.776			
Serum AFP (≤20 versus >20 ng/ml)	1.021	0.653-1.596	0.927				0.992	0.618-1.593	0.974			
Child-pugh score (A versus B)	0.984	0.654-1.481	0.940				1.091	0.705-1.691	0.695			
Tumor number (single versus multiple)	0.470	0.322-0.687	<0.001	0.982	0.501-1.923	0.958	0.442	0.297-0.659	<0.001	1.046	0.515-2.122	0.901
Maximal tumor size (≤5 versus >5 cm)	0.668	0.451-0.989	0.044	0.844	0.524-1.357	0.483	0.656	0.433-0.993	0.046	0.869	0.529-1.429	0.581
Tumor encapsulation (absent versus present)	2.373	1.622-3.472	<0.001	0.704	0.388-1.277	0.247	2.438	1.633-3.640	<0.001	0.637	0.340-1.192	0.158
Microvascular invasion (absent versus present)	0.434	0.297-0.635	<0.001	0.450	0.257-0.789	0.005	0.422	0.282-0.629	<0.001	0.433	0.243-0.772	0.005
Tumor differentiation (I-II versus III-IV)	0.453	0.304-0.673	<0.001	0.946	0.591-1.513	0.816	0.415	0.276-0.626	<0.001	0.869	0.536-1.408	0.567
TNM stage (I-II versus III)	0.125	0.082-0.190	<0.001	0.142	0.070-0.288	<0.001	0.114	0.074-0.176	<0.001	0.132	0.063-0.276	<0.001
**CAMSAP2** expression (negative versus positive)	0.433	0.295-0.636	<0.001	0.621	0.399-0.967	**0.035***	0.396	0.263-0.595	<0.001	0.593	0.370-0.952	**0.030***

Table S4. Correlation between EB1 expression and clinicopathological characteristics of HCC in human HCC tissues from two independent cohorts

Clinicopathological variables		Cohort I Tumor EB1 expression Negative (n=203)	Positive (n=157)	*P* Value	Cohort II Tumor EB1 expression Negative (n=100)	Positive (n=78)	*P* Value
Age		52.68(9.56)	51.31(9.18)	0.168	50.12(12.28)	52.09(10.88)	0.266
Sex	female	30	26	0.662	15	19	0.128
	male	173	131		85	59	
Serum AFP	≤20ng/ml	40	25	0.408	19	20	0.361
	>20ng/ml	163	132		81	58	
Child-pugh score	Class A	170	126	0.407	69	56	0.743
	Class B	33	31		31	22	
Tumor number	single	144	93	0.025	62	37	0.068
	multiple	59	64		38	41	
Maximal tumor size	≤5cm	107	69	0.111	43	29	0.446
	>5cm	96	88		57	49	
Tumor encapsulation	absent	51	58	0.020	35	46	0.002
	present	152	99		65	32	
Microvascular invasion	absent	128	76	0.007	62	35	0.024
	present	75	81		38	43	
Tumor differentiation	I-II	165	104	0.001	81	48	0.006
	III-IV	38	53		19	30	
TNM stage	I-II	164	108	0.010	81	48	0.006
	III	39	49		19	30	

Table S5. Correlation between Trio expression and clinicopathological characteristics of HCC in human HCC tissues from two independent cohorts

		Cohort I			Cohort II		
		Tumor Trio expression			Tumor Trio expression		
Clinicopathological variables		Negative (n=210)	Positive (n=150)	*P* Value	Negative (n=110)	Positive (n=68)	*P* Value
Age		52.44(9.21)	51.58(9.69)	0.392	50.73(11.94)	51.40(11.36)	0.712
Sex	female	33	23	1.000	23	11	0.577
	male	177	127		87	57	
Serum AFP	≤20ng/ml	36	29	0.677	26	13	0.577
	>20ng/ml	174	121		84	55	
Child-pugh score	Class A	170	126	0.487	81	44	0.239
	Class B	40	24		29	24	
Tumor number	single	148	89	0.032	63	36	0.642
	multiple	62	61		47	32	
Maximal tumor size	≤5cm	112	64	0.054	51	21	0.043
	>5cm	98	86		59	47	
Tumor encapsulation	absent	48	61	<0.001	41	40	0.006
	present	162	89		69	28	
Microvascular invasion	absent	132	72	0.007	69	28	0.006
	present	78	78		41	40	
Tumor differentiation	I-II	176	93	<0.001	86	43	0.038
	III-IV	34	57		24	25	
TNM stage	I-II	181	91	<0.001	87	42	0.016
	III	29	59		23	26	

Table S6. Correlation between Ace-tubulin expression and clinicopathological characteristics of HCC in human HCC tissues from two independent cohorts

		Cohort I			Cohort II		
		Tumor Ace-tubulin expression			Tumor Ace-tubulin expression		
Clinicopathological variables		Negative (n=188)	Positive (n=172)	P Value	Negative (n=98)	Positive (n=80)	P Value
Age		51.62(8.68)	52.59(10.14)	0.326	50.79(11.70)	51.23(11.76)	0.804
Sex	female	32	24	0.468	18	16	0.849
	male	156	148		80	64	
Serum AFP	≤20ng/ml	37	28	0.414	24	15	0.370
	>20ng/ml	151	144		74	65	
Child-pugh score	Class A	159	137	0.270	65	60	0.250
	Class B	29	35		33	20	
Tumor number	single	136	101	0.008	65	34	0.002
	multiple	52	71		33	46	
Maximal tumor size	≤5cm	104	72	0.012	41	31	0.759
	>5cm	84	100		57	49	
Tumor encapsulation	absent	37	72	<0.001	28	53	<0.001
	present	151	100		70	27	
Microvascular invasion	absent	133	71	<0.001	65	32	0.001
	present	55	101		33	48	
Tumor differentiation	I-II	158	111	<0.001	79	50	0.011
	III-IV	30	61		19	30	
TNM stage	I-II	160	112	<0.001	82	47	<0.001
	III	28	60		16	33	

Table S7. Correlation between HDAC6 expression and clinicopathological characteristics of HCC in human HCC tissues from two independent cohorts

Clinicopathological variables		Cohort I			Cohort II		
		Tumor HDAC6 expression			Tumor HDAC6 expression		
		Negative (n=215)	Positive (n=145)	*P* Value	Negative (n=102)	Positive (n=76)	*P* Value
Age		51.24(9.07)	53.34(9.79)	0.038	50.57(11.73)	51.54(11.70)	0.585
Sex	female	37	19	0.304	18	16	0.570
	male	178	126		84	60	
Serum AFP	≤20ng/ml	43	22	0.266	26	13	0.203
	>20ng/ml	172	123		76	63	
Child-pugh score	Class A	172	124	0.207	72	53	1.000
	Class B	43	21		30	23	
Tumor number	single	128	109	0.002	46	53	0.001
	multiple	87	36		56	23	
Maximal tumor size	≤5cm	98	78	0.134	37	35	0.218
	>5cm	117	67		65	41	
Tumor encapsulation	absent	79	30	0.001	59	22	<0.001
	present	136	115		43	54	
Microvascular invasion	absent	107	97	0.002	46	51	0.004
	present	108	48		56	25	
Tumor differentiation	I-II	146	123	<0.001	64	65	0.001
	III-IV	69	22		38	11	
TNM stage	I-II	145	127	<0.001	59	70	<0.001
	III	70	18		43	6	

Table S8. Primers used in the chromatin immunoprecipitation assay

Primer name	Primer sequences	Enzyme
Primers for HDAC6 promoter construct:		
(-1690/+136)HDAC6 sense:	5’-ATATACGCGTCCACTGAGACCGTATGTG-3’	*MluI*
(-1384/+136)HDAC6 sense:	5’-ATATACGCGTCAGGACATCTTCAAGAGG-3’	*MluI*
(-469/+136)HDAC6 sense:	5’-ATATACGCGTGACGACAGCGACGATAGC-3’	*MluI*
(-199/+136)HDAC6 sense:	5’-ATATACGCGTCAGTCGAGAGACGAGGCC-3’	*MluI*
Antisense:	5’-TATACTCGAGGACCGGTACCTTCCACTC-3’	*XhoI*
Primers for HDAC6 promoter site-directed mutagenesis:		
binding site 1 mutation sense:	5’-GGCGCGGCCTTactaCACGGTCCCCTC-3’	
binding site 1 mutation antisense:	5’-GAGGGGACCGTGtagtAAGGCCGCGCC-3’	

Table S9. Commercially available reagents used in this study

Reagent	Catalog #	Provider
Alpha Tubulin	Ab7291	Abcam
CAMSAP2	17880-1-AP	Proteintech
CAMSAP2	HPA026511	Atlas
CAMSAP2	Ab86683	Abcam
EB1	Ab53358	Abcam
EB1	17717-1-AP	Proteintech
Rac1	Ab33186	Abcam
Trio	HPA008157	Atlas
Trio	H00007204-A01	Abnova
Rac1 Pulldown Assay Kit	BK035-S	Cytoskeleton
Acetyl-α-Tubulin	5335	Cell Signaling
Acetylated Tubulin	66200-1-lg	Proteintech
HDAC6	sc-28386	Santa Cruz
HDAC6	56343	Novus Biologicals
AlphaTAT1	ARP42642_T100	Aviva Systems Biology
SIRT2	sc-28298	Santa Cruz
GM130	Ab52649	Abcam
Gamma-tubulin	Ab179503	Abcam
Phospho-JNK	4668	Cell Signaling
Phospho-c-Jun	3270	Cell Signaling
GAPDH	BM3876	Boster
ActinRed	KGMP0012	KeyGEN
Tubulin-Tracker Red	C1050	Beyotime
EHop-016	S7319	Selleck
ITX3	HY-16663	Medchemexpress
Tubacin	HY-13428	Medchemexpress
Thiomyristoyl	HY-101278	Medchemexpress
SP600125	S1460	Selleck
NSC23766	HY-15723	Medchemexpress
Goat Anti-Mouse IgG Light	A25021	Abbkine
Goat Anti-Rabbit IgG heavy	A25222	Abbkine
